# Catalyst Hide‐and‐Seek Beneath Porous Support Surfaces: Pinpointing Active Site Distribution Through Resonance Energy Transfer

**DOI:** 10.1002/anie.4042285

**Published:** 2026-05-23

**Authors:** Buddhima K. P. Maldeni Kankanamalage, William J. Thompson, Danielle N. Smith, Grace C. Thaggard, Namodhi Wijerathne, Isabella Incognito, Jeffery A. Byers, Jia Niu, Natalia B. Shustova

**Affiliations:** ^1^ Department of Chemistry and Biochemistry University of South Carolina Columbia South Carolina USA; ^2^ Department of Chemistry Boston College Chestnut Hill Massachusetts USA; ^3^ Department of Chemistry and Center for Catalysis University of Florida Gainesville Florida USA

**Keywords:** active sites, catalyst distribution, heterogeneous catalysis, metal‐organic frameworks, photophysics

## Abstract

Merging the high selectivity and efficiency of homogeneous catalysts with the recyclability of heterogeneous systems represents an attractive, industry‐driven concept that can be realized through the “heterogenization” of existing molecular catalysts by incorporating them into porous solid‐state matrices. The concept proposed herein uses Förster resonance energy transfer analysis to establish the first direct correlations among matrix topology, catalyst integration strategy, and active site positioning in porous materials without employing fluorescent model systems. This catalyst mapping method can be applied to several classes of porous materials, including metal‐organic frameworks and mesoporous silica. It addresses the existing challenges in relating the factors that control the spatial surface (re)distribution of molecular catalysts within such matrices before and after catalytic transformations. On the example of a series of six different catalyst‐integrated materials, Å‐level mapping of active site distribution was correlated with the nature of the porous host and the catalyst integration mechanism, which dictates the loading and accessibility of integrated catalysts. Thus, these studies provide a foundation for developing a framework to guide the design of recyclable heterogeneous catalysts with well‐defined active site distributions, both before and after catalytic transformations, which are key fundamental parameters for heterogeneous catalysis.

## Graphical abstract

Like a shark playing hide‐and‐seek in a coral reef, energy transfer (ET) finds hidden “fish” catalysts in the porous support “reef.” ET allows catalytic species to be precisely mapped, revealing whether they reside near the surface, deep in the pores, or are uniformly distributed. This approach ensures controlled catalyst distribution, highlighting how pore size and catalyst integration method govern catalyst positioning and accessibility.

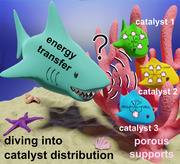

## 1 Introduction

Synergy between the advantages of heterogeneous catalytic systems (e.g., stability and recyclability) and the efficiency and selectivity of homogeneous catalysts can be achieved by integrating the latter into synthetically modular, porous platforms [1, 2, 3, 4, 5, 6, 7, 8, 9, 10, 11, 12, 13, 14, 15]. Furthermore, incorporation of industrially relevant molecular catalysts into porous supports (e.g., metal‐organic frameworks (MOFs) or mesoporous silica) [16, 17, 18, 19, 20, 21, 22, 23, 24, 25, 26, 27, 28, 29, 30, 31, 32, 33, 34, 35, 36, 37] could help shift industrial processes toward more sustainable “green” practices [38, 39, 40, 41, 42, 43, 44, 45, 46, 47, 48, 49]. However, accelerating progress in this direction requires a fundamental understanding of catalytic site distribution and its dependence on the choice of support, as well as on the synthetic strategy used to control site integration and accessibility within porous solid‐state matrices. It also requires insight into how these active sites are redistributed after catalytic transformations occur [24, 31, 32, 33, 36, 37, 40, 50, 51, 52, 53].

**SCHEME 1:**
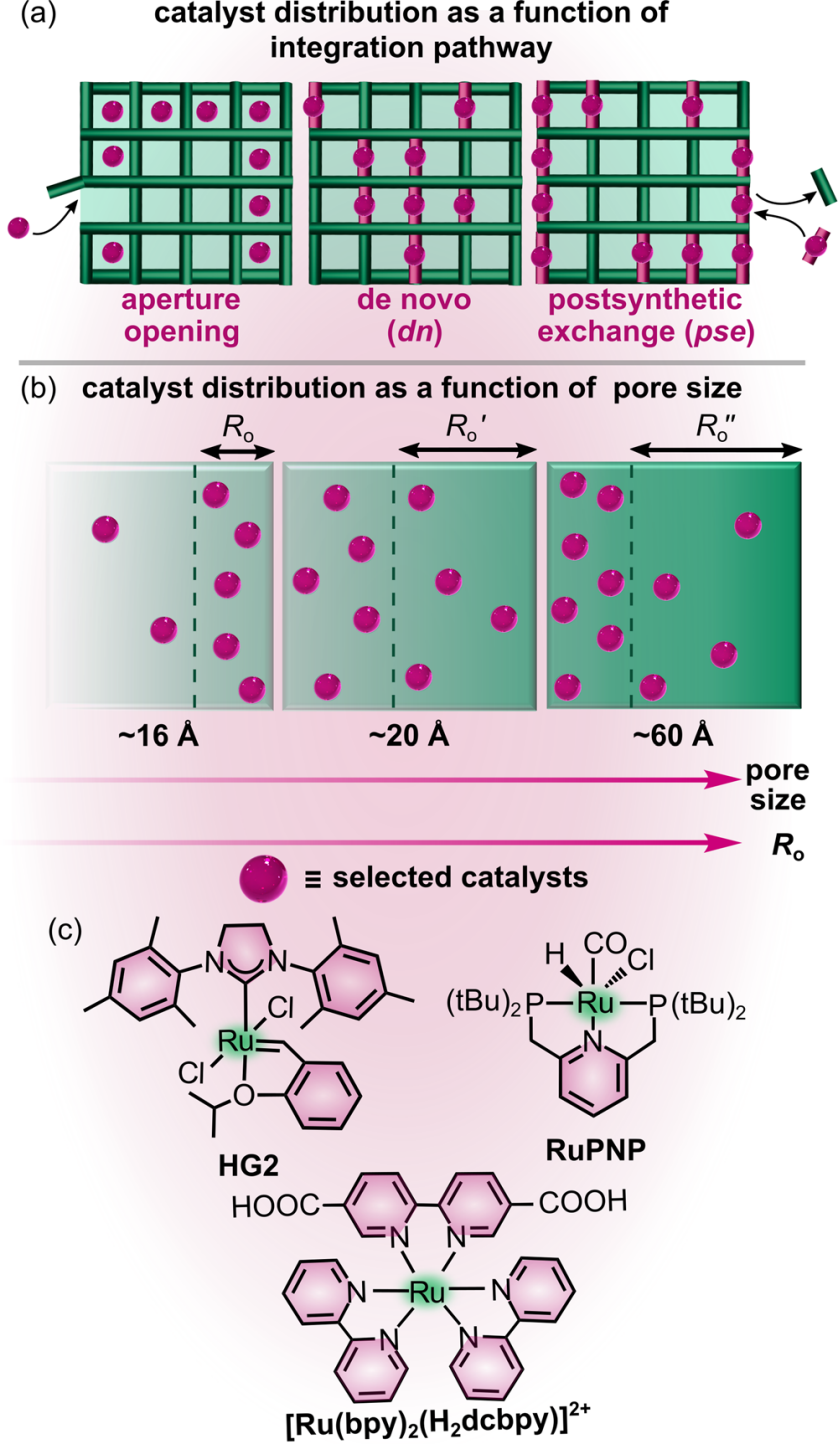
Schematic representation of (a) catalyst (pink spheres) distribution as a function of integration pathway and (b) catalyst distribution in various porous scaffolds (green blocks). A correlation between the scaffold pore size and Förster radius, *R*_o_, is shown by pink arrows. (c) Chemical structures of the selected catalysts: Hoveyda‐Grubbs second‐generation catalyst (HG2), (^tBu^PNP)Ru(CO)HCl (RuPNP), and carboxylic acid‐functionalized tris(2,2'‐bipyridyl)ruthenium(II) ([Ru(bpy)_2_(H_2_dcbpy)]^2+^).

The lack of fundamental knowledge on this topic is further exacerbated when accounting for the variety of possible mechanisms employed for catalyst integration in porous matrices and their effects on active site positioning and catalyst performance (Scheme 1).

The approaches for catalyst integration may include (i) aperture‐opening encapsulation (i.e., noncovalent integration of catalysts within MOF pores via linker association‐dissociation processes) [32, 33], (ii) catalyst diffusion, (iii) *de novo* (*dn*) synthesis in which the catalyst is introduced during framework assembly, and (iv) postsynthetic exchange (*pse*) of MOF linkers for catalytically active analogs [54, 55, 56, 57]. Thus, there is an urgent need for directly mapping how catalyst integration pathways and pore size/topology impact the spatial distribution of active sites in real heterogeneous catalytic systems under industrially relevant conditions, thereby laying the foundation for the rational design of solid‐supported catalysts. Despite the critical need for predictive tools to guide heterogeneous catalyst design, the previous limited attempts to map catalytically active sites within porous scaffolds have mainly relied on fluorescent tags or model systems, assuming that the used models and molecular catalysts possess similar characteristics [16, 35, 51, 58, 59, 60, 61, 62, 63, 64, 65, 66, 67, 68].

**FIGURE 1:**
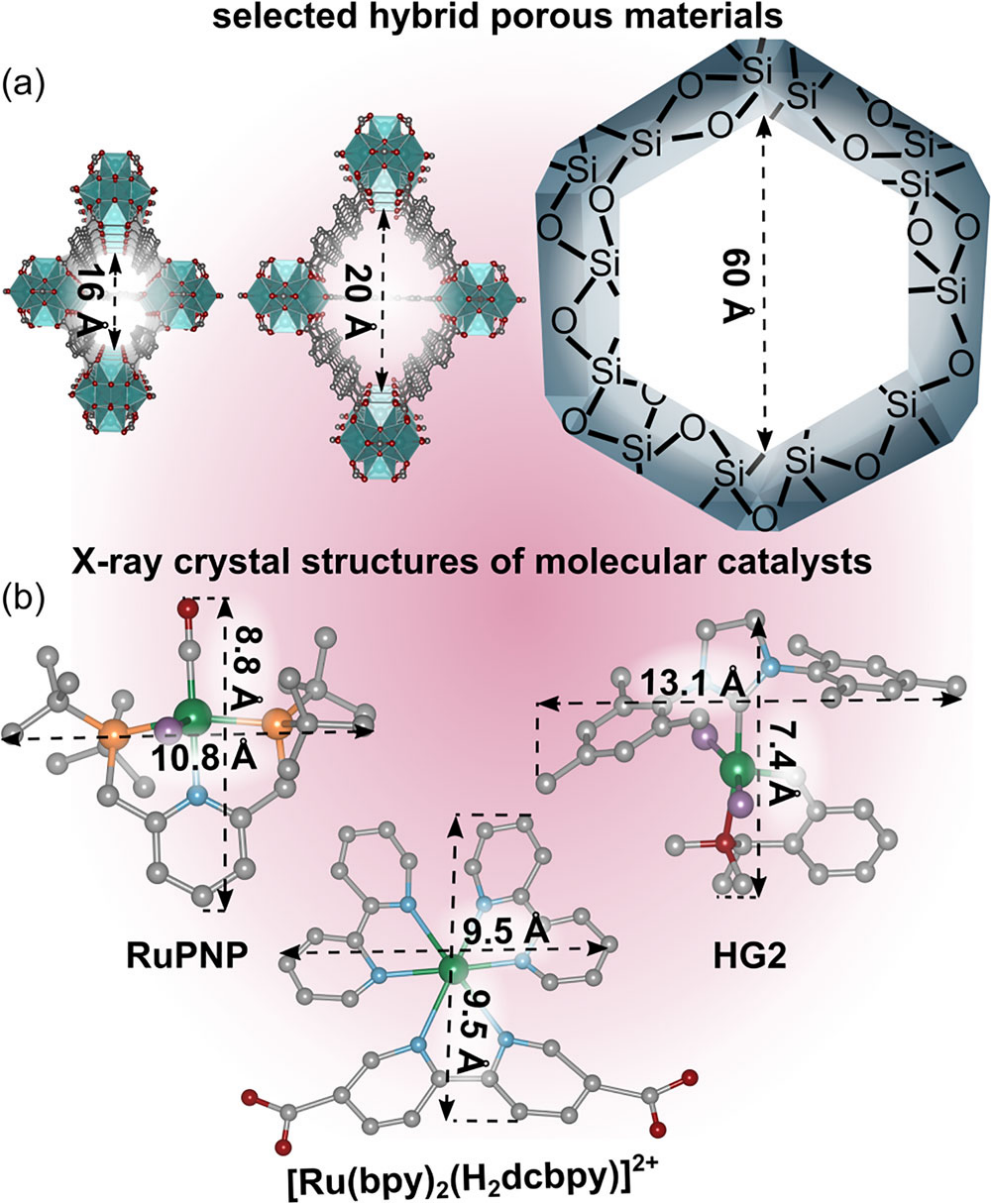
(a) The selected porous supports: UiO‐66 (left), UiO‐67 (middle), and SBA‐15 (right). (b) X‐ray crystal structures of RuPNP, [Ru(bpy)_2_(H_2_dcbpy)]^2+^, and HG2. Teal, gray, red, green, purple, orange, and blue spheres represent Zr, C, O, Ru, Cl, P, and N atoms, respectively. Hydrogen atoms and solvent molecules have been omitted for clarity.

**SCHEME 2:**
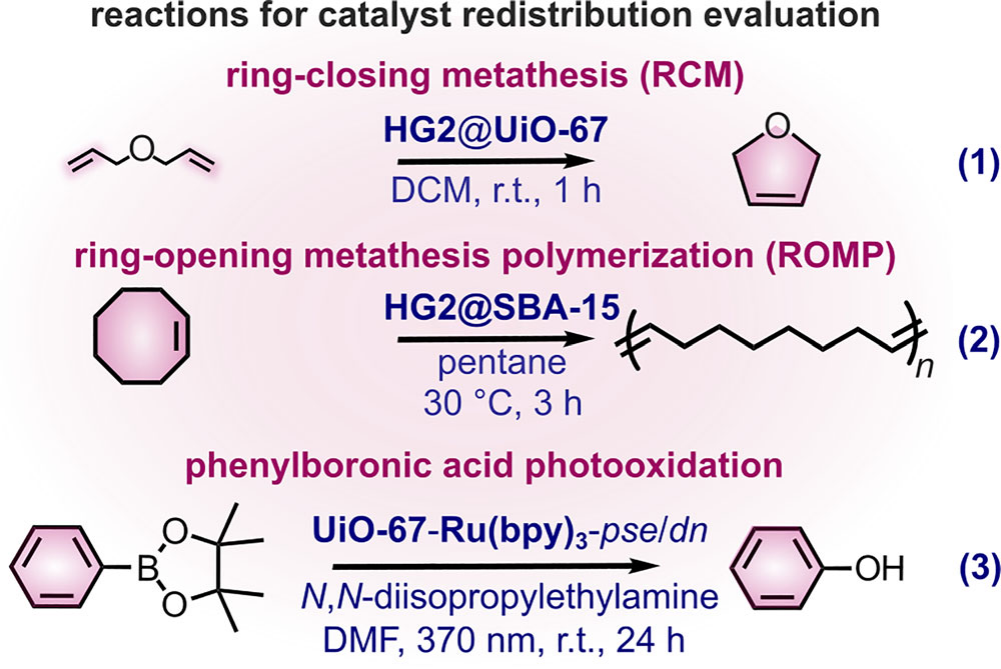
Reaction conditions for ring‐closing metathesis (RCM, (1) and ring‐opening metathesis polymerization (ROMP, (2) using the Hoveyda‐Grubbs second‐generation catalyst (HG2), as well as phenylboronic acid photooxidation using UiO‐67‐Ru(bpy)_3_‐*pse*/*dn* (3). The structures of the related catalysts are shown in Scheme 1 and Figure 1.

In these studies, we focus on real catalytic systems (using neither mimics nor fluorescent tags) and present a correlation among the type of porous support, the synthetic methodology for catalyst integration, and active site distribution that provides an avenue for predicting and tailoring catalyst distribution across distinct classes of porous materials. On the examples of six catalytic platforms based on MOFs and mesoporous silica as two distinct classes of porous supports, we applied Förster resonance energy transfer (FRET) studies to directly map active sites at the Å‐level. Specifically, we reveal how pore size and topology influence catalyst positioning upon increasing pore size from 16 to 60 Å in MOFs and mesoporous silica, yielding the only reported quantitative data on catalyst distribution across multiple material classes (Figure 1). This work is the only investigation of how four catalyst integration methods, such as aperture‐opening encapsulation, diffusion, *dn* synthesis, and *pse*, affect active site positioning within the porous matrix. We assessed potential catalyst migration under industrially relevant reaction conditions, including ring‐closing metathesis (RCM), ring‐opening metathesis polymerization (ROMP), and phenylboronic acid photooxidation (Schemes 1 and 2). Moreover, we unveil how host crystallinity influences catalyst (re)distribution in porous supports and can be used as an additional form of control over catalyst performance by selecting hosts with varying degrees of crystallinity (e.g., MOFs vs. silica). Overall, these studies provide critical insights into catalyst distribution in porous scaffolds and establish a direct correlation between support architecture and integration methodology, enabling rational design principles for the next‑generation hybrid heterogeneous catalytic systems with enhanced efficiency.

## 2 Results and Discussion

The presented work addresses the conceptual challenges in mapping Å‐level catalyst distribution in the outer layers of porous supports using three types of ruthenium‐based catalysts (vide infra) used for CO_2_ hydrogenation, phenylboronic acid photooxidation, RCM, and ROMP across six different platforms, as discussed below. More importantly, this work does not utilize any fluorescent tags or model systems and instead directly probes catalyst positioning in real, industrially relevant platforms. The selected porous platforms for catalyst integration span two classes of materials, MOFs and mesoporous silica, enabling integration of molecular catalysts through four distinct mechanisms: (1) aperture‐opening encapsulation relying on linker association–dissociation in metal‐organic systems [32, 33], (2) physisorption of molecular catalysts in porous hosts via diffusion [69], (3) postsynthetic exchange (*pse*) of MOF linkers for catalytically active analogs [54], and (4) *de novo* (*dn*) incorporation of catalysts occurring during solvothermal MOF synthesis [54, 55]. The schematic differences in the executed approaches 1, 3, and 4 are illustrated in Scheme 1. Notably, all four mechanisms are existing useful tools for the integration of catalytically active sites within porous scaffolds in the field of heterogeneous catalysis, and, therefore, the gained fundamental insights on the active site distribution can be applied toward the design of a wide variety of catalytic materials. The specific solid‐state platforms for catalyst integration were selected based on three criteria listed below: (i) pore diameters sufficient to accommodate the selected catalytically active molecular complexes, (ii) chemical stability of the porous support under selected encapsulation conditions, and (iii) compatibility with the mentioned suite of catalyst integration techniques (1–4, vide supra). Collectively, these criteria make certain classes of MOFs and mesoporous silica suitable heterogeneous supports capable of maintaining the catalytic performance of molecular compounds [24, 37, 50, 51, 70, 71, 72] while also offering ease of separation and recycling [19, 54, 72, 73, 74].

Among possible metal‐organic porous supports, Zr‐based MOFs, UiO‐66 (Zr_6_O_4_(OH)_4_(BDC)_6_, BDC^2–^ = benzene‐1,4‐dicarboxylate; UiO = University of Oslo) and UiO‐67 (Zr_6_O_4_(OH)_4_(BPDC)_6_, BPDC^2–^ = 1,4‐biphenyldicarboxylate), satisfy the listed (i–iii) criteria. For instance, UiO‐66 MOF (16‐Å pore size, diagonal) is suitable for the integration of (^tBu^PNP)RuHCl(CO) (RuPNP, ^tBu^PNP = 2,6‐bis((di‐*tert‐*butyl‐phosphino)methyl)pyridine) (8.8 × 10.8 Å) while both UiO‐66 and UiO‐67 (20‐Å pore size, diagonal) were used for the integration of Hoveyda–Grubbs second‐generation catalyst (HG2) (13.1 × 7.4 Å) [33, 50, 51, 75, 76, 77]. In addition to geometrical considerations, both frameworks, UiO‐66 and UiO‐67, are chemically stable toward selected catalytic reaction conditions (vide infra). For example, UiO‐66 is stable in a pH range of 3–9, maintaining its crystallinity in various organic solvents (e.g., tetrahydrofuran (THF), *N*,*N*‐dimethylformamide (DMF), dichloromethane (DCM), acetonitrile, toluene, and dimethyl sulfoxide) [33, 50, 51, 57, 75, 78, 79, 80, 81, 82, 83, 84]. Due to the isoreticular nature of UiO‐66 and UiO‐67 as well as the same synthetic approaches available for catalyst integration, the prepared catalytic systems allowed for a direct comparison of pore‐confinement effects on catalyst distribution. In addition, UiO‐67 was employed to probe how the synthetic methodology for catalyst integration could possibly affect catalyst distribution. The latter was shown through the incorporation of carboxylic acid‐functionalized catalyst, bis(2,2’‐bipyridine)(5,5’‐dicarboxy‐2,2’‐bipyridine)ruthenium(II) ([Ru(bpy)_2_(H_2_dcbpy)]^2+^), as linkers using a *pse* approach (3) and a *dn* strategy (4), as discussed below.

To extend the support scope beyond MOFs, we also selected SBA‐15, a mesostructured silica with significantly larger cylindrical mesopores (∼60 Å; Figure 1). SBA‐15 provides a contrast to MOF‐based heterogeneous catalysts, since its pore dimensions are fourfold larger than those in, for instance, UiO‐66 (vide supra) [37, 52, 69, 71, 76]. Furthermore, it has already been applied as a support in a wide range of catalytic reactions (e.g., olefin metathesis, C–C coupling, and petroleum refinement) [52, 85, 86, 87, 88] due to its large surface area, open architecture, and ability to incorporate catalytically active centers within its pores. The combination of selected scaffolds spans a wide range of pore sizes (e.g., 16–60 Å) and structural diversity from microporous frameworks to mesoporous materials [39, 50, 77]. Thus, the modularity of MOFs, particularly UiO‐66 and UiO‐67, allows for the employment of all integration approaches (1–4, vide supra) as well as precise tuning of internal voids, while SBA‐15 offers a complementary mesoporous scaffold used herein as a representative of porous, industrially relevant supports.

The evaluated molecular catalytic systems (Scheme 1 and Figure 1) include RuPNP, HG2, and a carboxylic acid‐functionalized tris(bipyridine)ruthenium(II) derivative ([Ru(bpy)_2_(H_2_dcbpy)]^2+^). The compounds RuPNP and Ru[(bpy)_2_(dcbpy)]Cl_2_ were synthesized based on known literature procedures [33, 54, 55, 89] while HG2 could be obtained commercially (synthetic details are provided in the Supporting Information (SI)).

The RuPNP@UiO‐66 system was initially selected as a point of reference because reliable procedures for RuPNP encapsulation in Zr‐MOFs are available, as are reported material characterization data obtained after catalyst integration [33, 51]. Encapsulation of RuPNP (8.8 × 10.8 Å) in the pores of UiO‐66 (7 × 12 × 16 (diagonal dimension) Å) was carried out using the aperture opening approach (1, vide supra) [33, 51]. For RuPNP@UiO‐66 preparation, a mixture of RuPNP and UiO‐66 was exposed to methanol at 55 °C for 24 h, after which the material underwent a literature‐based catalytic pretreatment step (exposure to 1,8‐diazabicyclo[5.4.0]undec‐7‐ene (DBU)/DMF under pressurized CO_2_ (3 bar) and H_2_ (37 bar) at 130°C for 45 min) to remove surface‐bound catalysts (a more detailed synthetic procedure is provided in the SI). The described RuPNP integration and catalytic pretreatment procedures were repeated twice per sample to increase the degree of incorporated catalyst, resulting in a loading of 0.005 wt% Ru based on inductively coupled plasma optical emission spectroscopy (ICP‐OES) analysis (see more details in the SI).

For the evaluation of catalyst distribution as a function of pore size (Scheme 1), we prepared HG2@UiO‐66 for comparison of the HG2 distribution within UiO‐67 and SBA‐15 platforms [50, 52, 69, 88]. The latter two possess much larger pores in comparison with UiO‐66. Thus, we studied the integration of the same HG2 catalyst within three different platforms: UiO‐66 (16 Å), UiO‐67 (20 Å), and SBA‐15 (60 Å). Integration of HG2 within UiO‐66 and UiO‐67 was carried out using reported aperture opening conditions (i.e., MOF powders were exposed to a 5.3 mM solution of HG2 in acetonitrile at room temperature for 72 h) [50], resulting in catalyst loadings of 0.060 and 0.10 wt% Ru for HG2@UiO‐66 and HG2@UiO‐67, respectively. The acquired loading confirms the hypothesis that the larger pore dimensions facilitated mass transport and greater guest uptake (e.g., a 1.7‐fold increase in catalyst loading in UiO‐67 compared to UiO‐66). It is important to note that RuPNP (8.8 × 10.8 Å) was not selected for integration in matrices with larger pore apertures (e.g., UiO‐67 (12 Å) and SBA‐15 (∼60 Å)) to prevent its potential leaching from the pores over time [33, 76].

Additionally, using a reported method, HG2 was integrated within SBA‐15 via diffusion by suspending SBA‐15 powder in a 1.1 mM solution of HG2 in DCM at room temperature for four hours, followed by the solvent removal under vacuum [52, 69], resulting in a catalyst loading of 0.16 wt% Ru. Importantly, immobilization of HG2 in SBA‐15 via diffusion (as opposed to aperture opening encapsulation in Zr‐MOFs, vide supra) is feasible due to its large pore size (∼60 Å) promoting mass transport, as well as the possibility for physisorption of HG2 to the silica‐based support.

After catalyst integration within RuPNP@UiO‐66, HG2@UiO‐66, HG2@UiO‐67, and HG2@SBA‐15 platforms, we probed how the catalyst distribution depends on the mechanism of encapsulation of catalytically active sites covalently attached to the linker, Ru[(bpy)_2_(dcbpy)]Cl_2_ on the example of UiO‐67‐Ru(bpy)_3_‐*pse* and UiO‐67‐Ru(bpy)_3_‐*dn* platforms. Notably, we refer to Ru[(bpy)_2_(dcbpy)]Cl_2_ as Ru(bpy)_3_ after integration within the MOF scaffold since the carboxylate groups are coordinated to the MOF backbone. These systems differ from each other in the catalyst integration procedure as described below. A [Ru(bpy)_2_(H_2_dcbpy)]^2+^ derivative was selected as a catalyst for the following integration within the porous scaffold, mainly based on two factors: its widespread use as a photocatalyst in photooxidation reactions and the possibility to modify the catalytic core with two carboxylic acid groups [54]. The latter factor enables catalyst installation via the postsynthetic linker exchange approach (*pse*, 3) and its integration during *de novo* MOF synthesis (*dn*, 4).

Preparation of UiO‐67‐Ru(bpy)_3_‐*pse* was carried out through postsynthetic exchange of the MOF linker, BPDC^2–^, with carboxylic acid‐functionalized Ru(bpy)_3_, achieved by heating a suspension of UiO‐67 and [Ru(bpy)_2_(H_2_dcbpy)]^2+^ at 85°C in a mixture of ethanol and water for 24 h, resulting in a catalyst loading of 0.161 wt% Ru. In contrast, the *dn* approach allows for simultaneous incorporation of both H_2_BPDC and [Ru(bpy)_2_(H_2_dcbpy)]^2+^ into the MOF structure during direct solvothermal synthesis, occurring at 120°C in DMF for 24 h (catalyst loading = 0.388 wt% Ru). Prior to further photophysical investigations, catalyst loadings for all materials were determined using ICP‐OES analysis, and all MOF‐based samples were analyzed by powder X‐ray diffraction (PXRD, Figures S26–S30) to ensure that their crystallinity was preserved throughout the experimental procedures.

**FIGURE 2:**
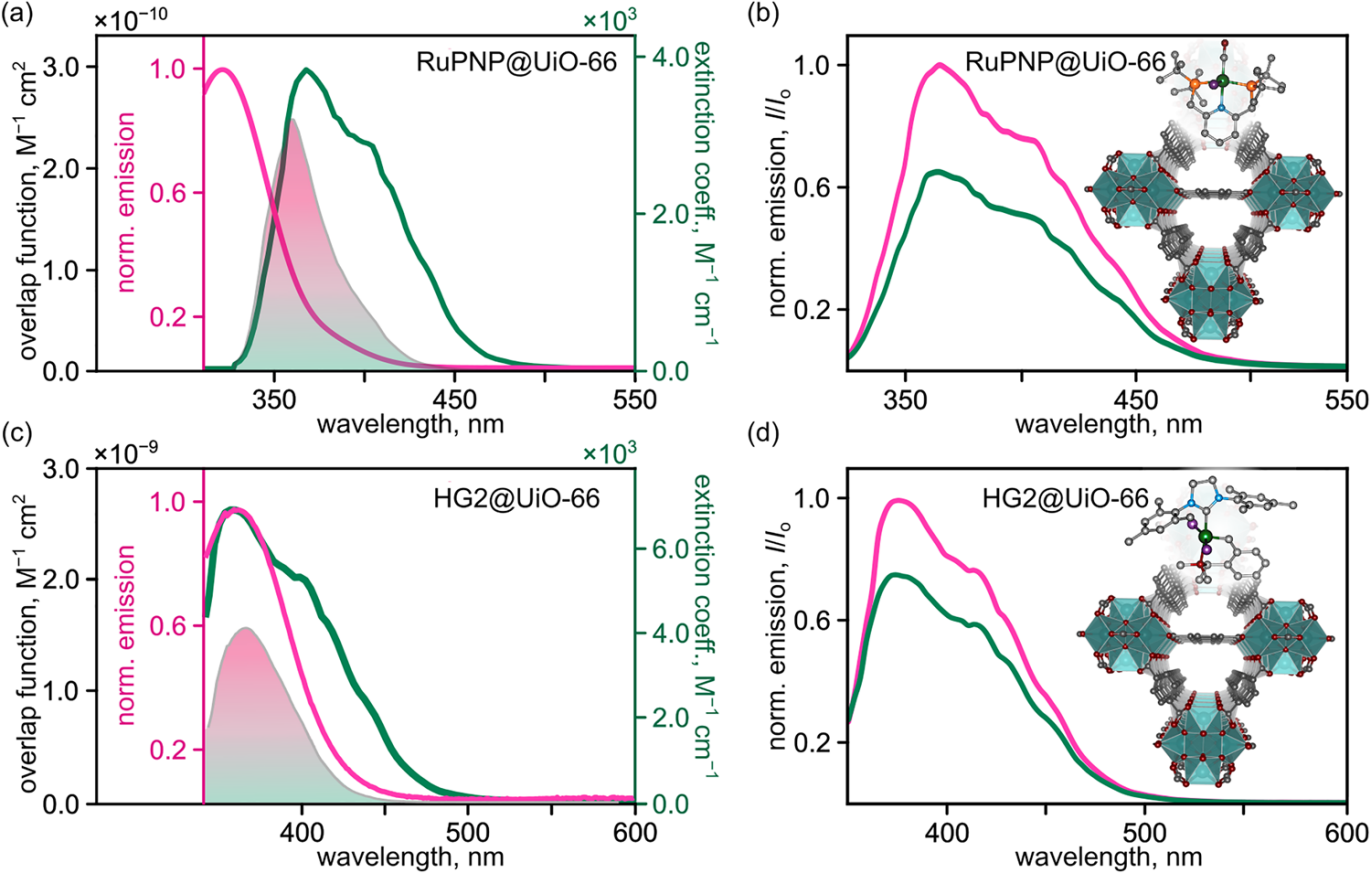
(a and c) Förster analysis of the FRET between PVK and either RuPNP or HG2 integrated within UiO‐66, illustrating the spectral overlap function (pink to green shade, left vertical axis), emission spectrum of PVK (pink line, arbitrary scale), and the molar extinction coefficient spectra (green line, right vertical axis) of RuPNP (a) and HG2 (c). Data for analysis of RuPNP@UiO‐66 were collected using THF solutions of PVK (4.0 µM, *λ*_ex_ = 310 nm) and RuPNP (90 µM). Data for analysis of HG2@UiO‐66 were collected using DCM solutions of PVK (15 µM, *λ*_ex_ = 345 nm) and HG2 (29 µM). (b and d) Emission spectra of PVK (pink line) and PVK in suspensions of either RuPNP@UiO‐66 (*b*; THF, *λ*_ex_ = 310 nm) or HG2@UiO‐66 (*d*; DCM, *λ*_ex_ = 345 nm; green line). The insets show schematic representations of the corresponding molecular catalysts integrated within UiO‐66. Teal, gray, red, green, purple, orange, and blue spheres represent Zr, C, O, Ru, Cl, P, and N atoms, respectively. H atoms and solvent molecules were omitted for clarity.

After the synthesis and proper characterization of the catalytically active systems described above, we employed FRET analysis to map catalyst distribution at the Å scale, primarily on the surface of heterogeneous porous materials. In general, for FRET to take place, a suitable donor‐acceptor pair with sufficient spectral overlap between the donor emission and the acceptor absorbance profiles should be selected. In our systems, the integrated catalysts act as acceptors exhibiting absorbance profiles in the 330–450 nm range, allowing us to extend the presented conceptual approach to catalyst mapping to any catalytic system, even those with very low fluorescence quantum yields. At the same time, poly(9‐vinylcarbazole) (PVK) can serve as a donor due to its relatively high photoluminescence quantum yield and emission profile compatible with the catalyst absorbance [90, 91, 92, 93]. Specifically, PVK emits strongly at ∼377 nm (*λ*_ex_ = 310–350 nm), which overlaps with the absorbance profiles of the selected catalysts (Figure 2 and Figures S1–S12), allowing FRET to take place. As an additional advantage, PVK does not penetrate the internal pore space of MOFs or mesoporous silica due to geometric constraints (*M*_n_ = 23000 by gel permeation chromatography and an estimated radius of gyration of ∼140 Å in toluene) [94, 95, 96, 97], ensuring that PVK remains surface‐localized. The control experiments that supported this conclusion were carried out prior to the photophysical analysis discussed below (Figures S14, S16, S18, S20, S22, and S24).

As stated above, initial studies began with the RuPNP@UiO‐66 system in order to evaluate the percentage of catalyst (acceptor) molecules in proximity to the surface‐limited donor molecules and correlate this outcome with the catalyst distribution within the porous matrix. As a first step, we calculated the spectral overlap function, *J*, between the emission of PVK in solution (donor) and the absorbance of catalysts (acceptor) according to the following equation: *J* = ∫*f*(*λ*)d*λ*, *f*(*λ*) = *F*(*λ*)*ε*(*λ*)*λ* ^4^ (where *F*(*λ*) is the emission spectrum of the donor (PVK)). The molar extinction coefficient *ε*(*λ*) spectrum of the acceptor (catalysts) was measured for the catalysts in solution in the relevant catalytic solvent (e.g., THF for CO_2_ hydrogenation, DCM for RCM, pentane/toluene for ROMP, and DMF for photooxidation reactions) to mimic the reaction environment (Figures S5–S9). The *R*_o_ values were estimated by using *R*_o_ (nm) = (8.79 × 10^−25^ × *κ* ^2^ *n* ^−4^ *Q*_d_ *J*)^1/6^ (where *Q*_d_ is the quantum yield of PVK in the same solvent used for determining *ε*(*λ*) of the acceptor, *κ* ^2^ is an orientation factor approximated to 2/3, and *n* is the refractive index approximated to 1) [98]. To summarize, the value of *R*_o_ is impacted by the average quantum yield of the donor (measured in triplicate for each sample), the emission profile of the donor in solution, and the molar extinction coefficient spectrum of the acceptor (catalyst) in solution.

In addition to the experiments carried out on the UiO‐66 system, we also performed several control experiments using the parent porous supports (i.e., without integrated catalysts) to ensure that PVK emission is not significantly quenched by the parent scaffolds themselves. In these experiments, the emission spectrum of PVK in the presence of suspended frameworks was collected.

Notably, we detected ∼3%–5% quenching of PVK emission in the presence of a UiO‐67 suspension due to the absorbance spectrum of UiO‐67 extending slightly into the visible region (Figures S18, S22, and S24). Under these conditions, the observed quenching of PVK emission in the presence of UiO‐67 was dependent on the exact molar ratio of PVK to UiO‐67. Therefore, we have repeated the control experiment (fluorescence quenching in the presence of parent solid‐support) for every FRET analysis of catalyst‐integrated UiO‐67 systems. The acquired emission quenching values due to parent UiO‐67 interference were corrected for catalyst‐integrated UiO‐67 samples (vide infra) to more accurately estimate the catalyst's contribution to FRET processes. Indeed, we detected no significant emission quenching for UiO‐66 and SBA‐15, supporting the fact that integrated catalysts play an essential role in promoting FRET processes (Figures S14, S16, and S20).

To map the catalyst distribution within RuPNP@UiO‐66, the material was suspended in a PVK/THF solution (the same solvent used for CO_2_ hydrogenation, vide infra), and the emission spectrum of the suspension was collected (Figure 2). To account for potential changes in PVK emission quenching due to differences in catalyst loading across platforms, the acceptor‐to‐donor molar ratio was systematically varied with catalyst loading until maximum FRET quenching was achieved (see SI for details). As a result, comparison of *E* values across all presented systems is independent of catalyst loading.

For the RuPNP@UiO‐66 system, a maximum fluorescence quenching of 36% was observed at an acceptor‐to‐donor molar ratio of 0.15 (see SI for details regarding calculation of the molar ratio), with a PVK concentration of 4.0 µM (*λ*_ex_ = 310 nm), as shown in Figure 2. The observed 36% quenching of PVK emission indicates that a significant fraction of the RuPNP molecules reside within the Förster critical radius of 1.7 nm from the MOF surface. This value suggests that encapsulated RuPNP molecules are preferentially localized near the surface in proximity to the external donor, PVK, enabling FRET (Figure 2). This example illustrates that the proposed concept of using FRET as a “ruler” to measure catalyst distribution within the outer layers of a porous host matrix can be applied to a real, catalytically active system by carefully selecting an appropriate donor‐acceptor pair.

**FIGURE 3:**
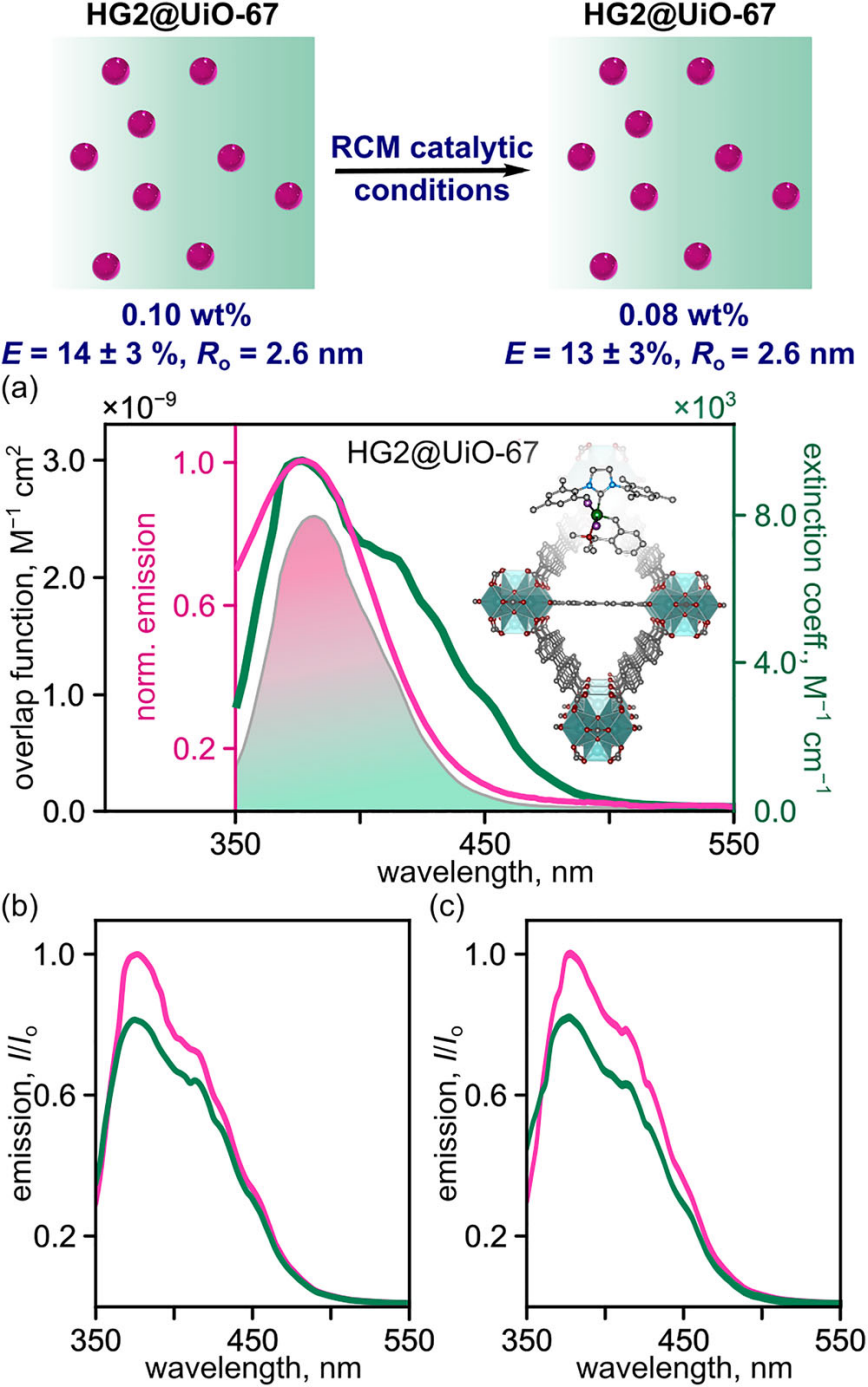
(top) Schematic representation showing no catalyst re‐distribution or leaching upon exposure of HG2@UiO‐67 to RCM catalytic conditions. (a) Förster analysis of ET between PVK and HG2 illustrating the spectral overlap function (pink to green shade, left vertical axis) calculated using the emission spectrum of PVK (pink line, arbitrary scale; 8.1 µM in DCM, *λ*_ex_ = 345 nm) and the molar extinction coefficient spectrum of HG2 (2.0 µM in DCM, green line, right vertical axis). The inset shows a schematic representation of the HG2 encapsulated in UiO‐67. Teal, gray, red, green, purple, and blue spheres represent Zr, C, O, Ru, Cl, and N atoms, respectively. H atoms and solvent molecules were omitted for clarity. (b and c) The emission spectra of PVK (pink lines) and the emission spectra of PVK in an HG2@UiO‐67 suspension (8.1 µM PVK in DCM, green line) before (b) and after (c) its exposure to RCM catalytic conditions.

**FIGURE 4:**
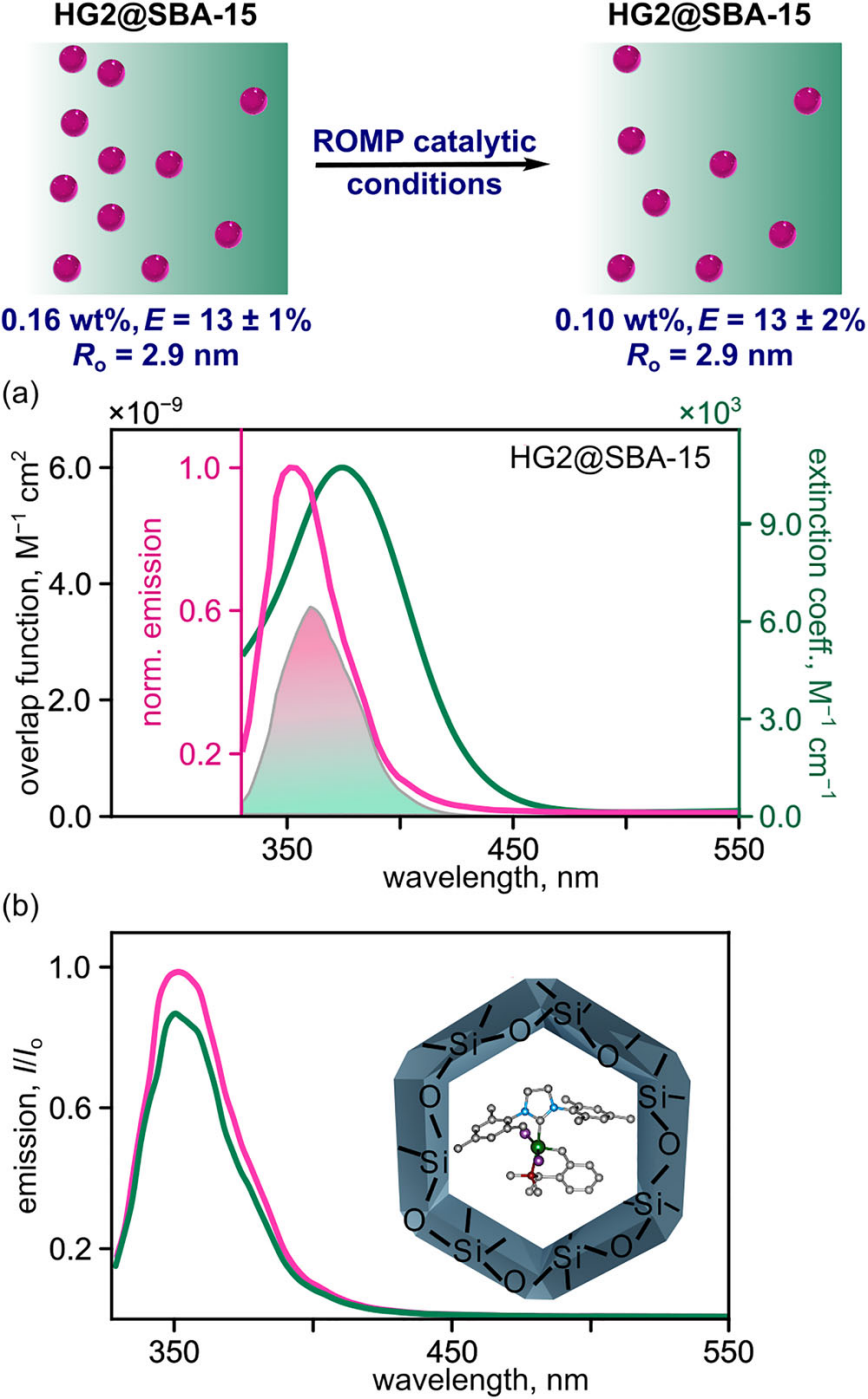
(*top*) Schematic representation showing minor changes in catalyst distribution in HG2@SBA‐15 after its exposure to ROMP catalytic conditions. (a) Förster analysis of ET between PVK and HG2 illustrating the spectral overlap function (pink to green shade, left vertical axis) calculated using the emission spectrum of PVK (pink line, arbitrary scale; 132 µM, *λ*_ex_ = 310 nm) and the molar extinction coefficient spectrum of HG2 (32 µM in pentane/toluene (9:1, v/v), green line, right vertical axis). (b) The emission spectrum of PVK (132 µM in DCM, *λ*_ex_ = 310 nm; pink line) and the emission spectrum of PVK in an HG2@SBA‐15 suspension (132 µM PVK in pentane/toluene (9:1, v/v), green line). The inset shows a schematic representation of HG2 encapsulated in SBA‐15. Gray, red, green, purple, and blue spheres represent C, O, Ru, Cl, and N atoms, respectively.

**FIGURE 5:**
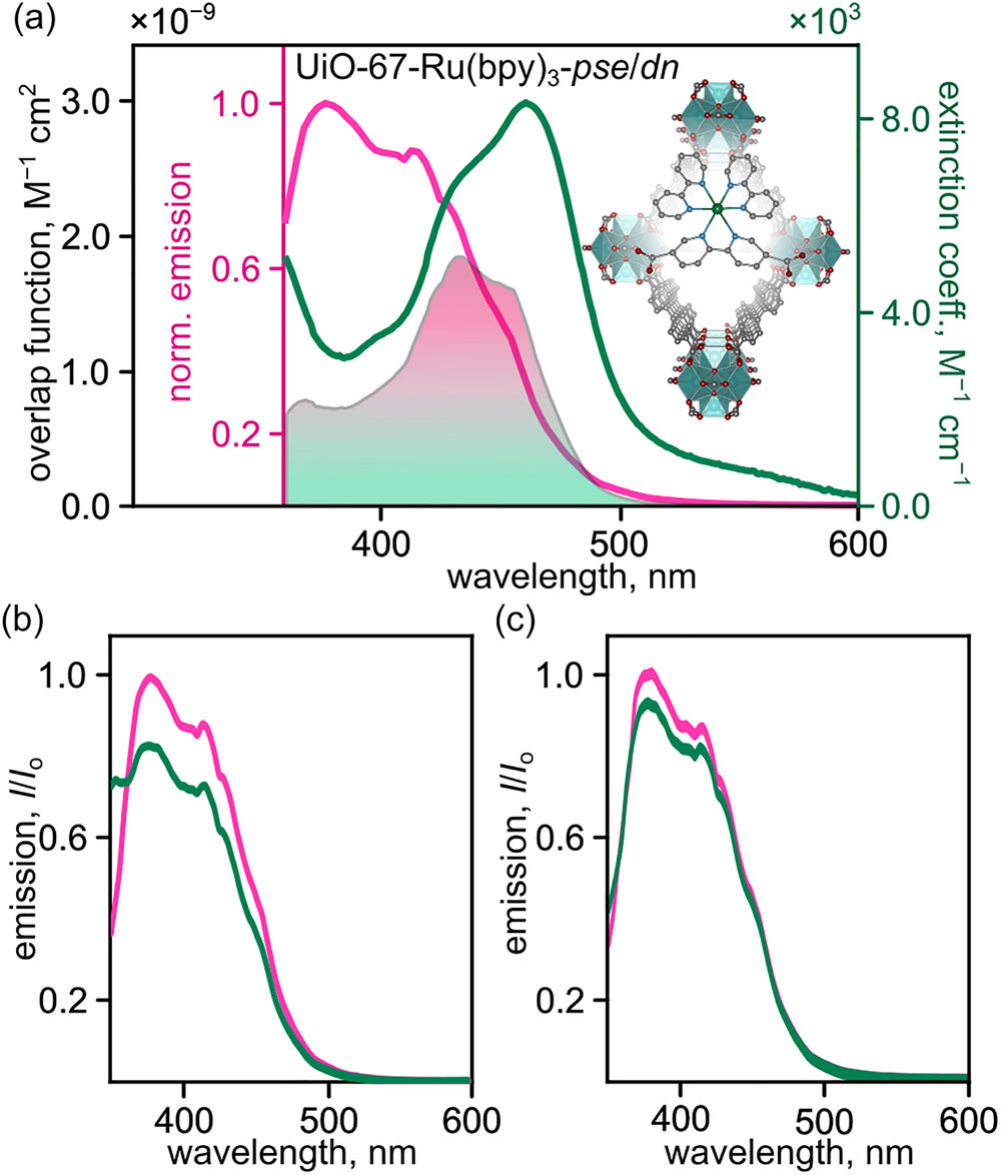
(a) Förster analysis of the ET between PVK and [Ru(bpy)_2_(H_2_dcbpy)]^2+^, illustrating the spectral overlap function (pink to green shade, left vertical axis) calculated using the emission spectrum of PVK (pink line, arbitrary scale; 5.6 µM, *λ*_ex_ = 345 nm) and the molar extinction coefficient spectrum of [Ru(bpy)_2_(H_2_dcbpy)]^2+^ (25 µM in DMF, green line, right vertical axis). The inset shows a schematic representation of [Ru(bpy)_2_(H_2_dcbpy)]^2+^ incorporated into UiO‐67. Teal, gray, red, green, and blue spheres represent Zr, C, O, Ru, and N atoms, respectively. H atoms and solvent molecules were omitted for clarity. (b) The emission spectrum of PVK (5.6 µM in DMF, *λ*_ex_ = 345 nm; pink line) and the emission spectrum of PVK in an UiO‐67‐Ru(bpy)_3_‐*pse* suspension (5.6 µM PVK in DMF, green line). (c) The emission spectrum of a PVK (4.2 µM in DMF, *λ*_ex_ = 345 nm; pink line) and the emission spectrum of PVK in an UiO‐67‐ Ru(bpy)_3_‐*dn* suspension (4.2 µM PVK in DMF, green line).

As a next step, we tested the catalyst distribution as a function of pore size in HG2@UiO‐66, HG2@UiO‐67, and HG2@SBA‐15 systems (Scheme 2). Similar to the procedure described above, all porous systems were suspended in PVK solutions, and the resulting fluorescence spectra are shown in Figures 2, 3, 4, 5. The summary of the photophysical data, including the used organic solvent and donor/acceptor concentrations, is also provided in Table 1 and Table S1. The control experiments demonstrated that fluorescence quenching due to the parent support was ≤1% for UiO‐66 and SBA‐15, whereas UiO‐67 resulted in *E* = 5 ± 3% (Figures S14, S16, S18, S20, S22, and S24).

**TABLE 1:** Summary of results for FRET measurements.

system	*J*, × 10^−14^ M^−1^cm^3^	*R*_o_, nm	*E*, %	solvent
RuPNP@UiO‐66	0.1	1.7 ± 0.05	36 ± 1	THF
HG2@UiO‐66	0.96	2.5 ± 0.07	23 ± 4	DCM
HG2@UiO‐67	1.3	2.6 ± 0.04	14 ± 3	DCM
HG2@SBA‐15	1.5	2.9 ± 0.04	13 ± 1	pentane/ toluene (v/v, 9:1)
UiO‐67‐Ru(bpy)_3_‐*pse*	1.5	2.6 ± 0.01	12 ± 2	DMF
UiO‐67‐Ru(bpy)_3_‐*dn*	1.5	2.6 ± 0.01	5 ± 3	DMF

For HG2@UiO‐66 and HG2@UiO‐67 systems, the quenching studies were conducted in DCM to provide an environment that mimics selected catalytic reaction conditions (vide infra). In the case of HG2@SBA‐15, measurements were carried out in a pentane/toluene (v/v, 9:1) solvent mixture since pentane served as the primary medium to replicate catalytic conditions, while the small fraction of toluene was included to enhance the solubility of the PVK donor. A reduction in photoluminescence intensity of PVK was observed in the presence of the HG2@UiO‐66, HG2@UiO‐67, and HG2@SBA‐15 suspensions with a maximum *E* of 23 ±  4% (Figure 2), 14  ±  3% (Figure 3), and 13  ±  1% (Figure 4), respectively, with the optimal acceptor‐to‐donor ratios of 0.19 (HG2@UiO‐66), 0.25 (HG2@UiO‐67), and 0.11 (HG2@SBA‐15). As described previously, differences in catalyst loading across all systems were accounted for by adjusting the concentration of the used PVK solutions based on the catalyst loading determined by ICP‐OES analysis. The estimated *R*_o_ values are provided in Table 1 and Figures 2, 3, 4. A comparison of the determined *R*_o_ and *E* values across all three systems, HG2@UiO‐66, HG2@UiO‐67, and HG2@SBA‐15, reveals that increasing pore size results in migration of catalytic centers away from the particle surface. Specifically, the relatively low value of *E* for HG2@SBA‐15 (13%) aligns with fewer catalyst molecules residing within *R*_o_ = 2.9 nm from the particle surface compared to, for instance, HG2@UiO‐66 (23%, *R*_o_ = 2.5 nm). This implies deeper diffusion of HG2 into the larger pores of HG2@SBA‐15 in comparison with UiO‐66 or UiO‐67 frameworks. These findings underscore how support topology, particularly pore size, may play a role in determining catalyst distribution and align with our hypothesis that increasing pore size facilitates deeper catalyst diffusion into the porous matrix. For more in‐depth studies of the active site distribution throughout the catalytic material lifecycle, we also considered that exposure of any heterogeneous catalytic platforms, such as HG2@UiO‐67, to catalytic reaction conditions could potentially induce redistribution of integrated catalysts, which may impact catalyst recyclability. To address this question, we investigated the HG2@UiO‐67 and HG2@SBA‐15 materials after exposure to the RCM and ROMP reaction conditions, respectively. For instance, catalyst distribution within HG2@UiO‐67 was evaluated after its use in the RCM catalytic reaction (Figure 3, Tables S4, S8, and Scheme 2 (1)) of allyl ether in DCM at room temperature. Photophysical analysis using the discussed FRET approach showed that, after exposure to the reaction conditions, the value of *E* decreased only slightly from 14 ± 3% to 13 ± 3%, and the Förster critical radius remained at 2.6 nm (Figure 3).

In addition, we evaluated catalyst loading before and after exposure to the RCM reaction conditions by ICP‐OES analysis and found no statistically significant changes in catalyst content. The minimal change in *E* combined with a consistent catalyst loading suggests minimal catalyst redistribution, affirming that the RCM process does not significantly alter catalyst positioning in HG2@UiO‐67.

In the case of HG2@SBA‐15, the material was subjected to the experimental conditions suitable for ROMP of *cis*‐cyclooctene (Scheme 2 (2)). The reaction was carried out in pentane at 30°C for three hours, resulting in high conversion (>99%), which is in line with literature reports [52, 69, 74, 87, 88] (Scheme 2 (2) and Figure 4). Based on the applied FRET analysis, exposure of HG2@SBA‐15 to catalytic conditions did not result in a significant change in ET efficiency (*E* = 13 ± 1% and 13 ± 2% before and after reaction, respectively), and *R*_o_ stayed the same at 2.9 nm. However, ICP‐OES data collected before and after exposure of HG2@SBA‐15 to ROMP conditions revealed a small but statistically significant decrease in catalyst loading (Figure 4; Tables S5 and S8). One of the possible explanations for these changes is that interior catalytic sites (i.e., HG2 molecules located far from the particle surface) underwent migration toward the particle surface, while there is a possibility of leaching a small number of surface‐bound catalysts. As a result, the total number of catalytic sites (acceptors) within *R*_o_ was relatively consistent, but the overall catalyst loading slightly decreased after reaction completion. Thus, the larger ∼60‐Å pores of SBA‐15 combined with the catalyst integration method (relatively weak physisorption of catalysts through diffusion) could potentially allow for slight catalyst migration upon exposure to relevant reaction conditions. In contrast, the smaller pores of UiO‐67 combined with the aperture‐opening catalyst installation method seems to promote catalyst retention.

As a final set of experiments, we probed whether the proposed FRET approach for mapping catalyst distribution could be applied to evaluate systems in which catalytically active moieties, such as Ru(bpy)_3_, are integrated into the same platform by two distinct approaches. We hypothesized that the *pse* approach would result in a preferential positioning of [Ru(bpy)_2_(H_2_dcbpy)]Cl_2_ toward the particle surface due to ligand exchange primarily occurring near the surface of the MOF. In contrast, the *dn* method was expected to result in a more uniform catalyst distribution as H_2_BPDC and [Ru(bpy)_2_(H_2_dcbpy)]^2+^ should be incorporated into the MOF at similar rates (Scheme 1). To probe this hypothesis, the UiO‐67‐Ru(bpy)_3_‐*pse* sample (catalyst loading of 0.161 wt% Ru) was suspended in a PVK solution in DMF. As expected, the photoluminescence intensity of PVK (*λ*_ex_ = 345 nm) decreased in the presence of UiO‐67‐Ru(bpy)_3_‐*pse* in comparison with that of pristine PVK solution emission (*λ*_em(max)_ = 377 nm). As a result, the value of *E* measured for PVK in the presence of UiO‐67‐Ru(bpy)_3_‐*pse* was found to be 12 ± 2% (taking into account ∼3% observed quenching due to parent UiO‐67; Figures 5 and S22; Tables S6 and S8). Similar to the systems discussed above, UiO‐67‐Ru(bpy)_3_‐*pse* was then exposed to phenylboronic acid photooxidation catalytic conditions (Scheme 2 (3)). As anticipated due to coordinative integration of the catalyst, the *E* value remains the same at 12 ± 3%, suggesting that the [Ru(bpy)_2_(H_2_dcbpy)]^2+^ centers do not undergo re‐distribution during the catalytic reaction.

Similar to UiO‐67‐Ru(bpy)_3_‐*pse*, the UiO‐67‐Ru(bpy)_3_‐*dn* sample was also suspended in a PVK solution in DMF. The estimated value of *E* for PVK in the presence of UiO‐67‐Ru(bpy)_3_‐*dn* was 5 ± 1% (taking into account ∼3% quenching due to the UiO‐67 support, Figures 5 and S24; Tables S7 and S8). Although the as‐synthesized UiO‐67‐Ru(bpy)_3_‐*dn* sample had a higher [Ru(bpy)_2_(H_2_dcbpy)]Cl_2_ loading (0.388 wt% Ru, 11 nmol as used in photoluminescence quenching experiment) compared to the UiO‐67‐Ru(bpy)_3_‐*pse* sample (0.161 wt% Ru, 4.1 nmol), it demonstrated lower energy transfer efficiency (5 ± 1% (*dn*) versus 12 ± 3% (*pse*)). Assuming a direct correlation between the amount of catalyst within the Förster radius (2.6 nm) and ET efficiency, we could infer that more [Ru(bpy)_2_(H_2_dcbpy)]^2+^ molecules in the *pse* sample are located near the MOF surface, resulting in a higher local concentration of acceptor molecules within *R*_o_ and thus more efficient FRET. This observation aligned with our hypothesis that the majority of the catalyst would be located at the UiO‐67‐Ru(bpy)_3_‐*pse* surface due to the postsynthetic exchange procedure, while a more even distribution of the catalyst would be achieved in the case of UiO‐67‐Ru(bpy)_3_‐*dn* due to simultaneous incorporation of linker and catalysts via a *de novo* approach. The preferential positioning of catalysts in UiO‐67‐Ru(bpy)_3_‐*pse* was further confirmed by confocal microscopy which showed bright emission at the edges of UiO‐67‐Ru(bpy)_3_‐*pse* crystals arising from the Ru(bpy)_3_ moieties (Figure S37).

## 3 Conclusion

In the presented studies, we investigated real catalytic systems without employing mimics or fluorescent tags for the first time, establishing direct correlations among the type of porous support, the synthetic methodology for catalyst integration, and the resulting active site distribution in the surface layer of a host matrix. This approach provides a framework for predicting and tailoring catalyst (re)distribution across distinct classes of porous materials prior to and after catalytic transformations. Using six catalytic platforms constructed with MOFs and mesoporous silica, we applied FRET studies as a noninvasive, generalizable mapping tool to directly map active sites with Å‐level precision. These studies also revealed how pore size and topology influence catalyst positioning as the pore diameter increases. Using the examples of two distinct classes of materials (i.e., Zr‐based MOFs and mesoporous silica SBA‐15), we showed that increasing pore size from 16 to 60 Å promotes deeper catalyst penetration within the bulk of porous materials, resulting in active sites positioned further from the particle surface, yielding the first quantitative comparison of catalyst distributions across multiple material classes.

Moreover, we provided proof‐of‐concept for using four common catalyst integration strategies, such as aperture‐opening encapsulation, diffusion, *dn*, and *pse*, which can be used to rationally target specific active site distribution within the surface layer of a solid support to achieve the desired catalytic activity. For instance, we present direct experimental confirmation that both aperture opening encapsulation and *pse* of organic linkers for catalytically active analogs result in surface‐localized active sites. In contrast, *dn* synthetic approaches are shown to yield a uniform distribution of active sites by anchoring catalysts throughout the material during framework formation. As a result, a higher loading of active sites can be achieved through *dn* synthesis, making it an advantageous approach for the design of catalysts targeting smaller molecules exhibiting facile diffusion throughout the matrix. Importantly, no catalyst redistribution in MOF‐based catalysts was observed under relevant catalytic conditions such as RCM and phenylboronic acid photooxidation, while also resulting in high reaction conversion, underscoring the future recyclability and high efficiency of these systems. Notably, investigations of the impact of catalyst positioning on overall catalytic activity (e.g., the relationship between catalyst distribution and turnover number or turnover frequency) are being actively pursued. For instance, catalyst penetration depth, pore geometry, and particle size likely govern the catalytic efficiency of a material, highlighting an opportunity to extend this research in the future. Finally, the presented studies showcase how the selection of the porous support and catalyst integration mechanism results in completely different catalyst distributions and loadings, ultimately defining their applicability as heterogeneous catalysts for CO_2_ reduction, RCM, ROMP, or phenylboronic acid photooxidation. The novel trends developed herein can be used to predict and target desirable catalyst behavior in solid‐state supports, enabling the rational design of efficient and recyclable heterogeneous catalysts for the upcoming era of sustainable chemical industry.

## Author Contributions

**Jeffery A. Byers**: conceptualization, supervision, project administration, funding acquisition. **Isabella Incognito**: investigation. **Buddhima K. P. Maldeni Kankanamalage**: writing ‐ original draft, writing – review and editing, investigation, formal analysis, visualization, methodology. **Jia Niu**: conceptualization, funding acquisition, project administration, resources, supervision, writing – review and editing. **Natalia B. Shustova**: conceptualization, funding acquisition, writing ‐ original draft, writing – review and editing, project administration, resources, supervision, methodology, formal analysis. **Danielle N. Smith**: visualization, writing – review and editing, investigation. **Grace C. Thaggard**: writing – original draft, writing – review and editing, visualization. **William J. Thompson**: investigation, formal analysis, writing – review and editing. **Namodhi Wijerathne**: resources, investigation.

## Conflicts of Interest

The authors declare no conflict of interest.

## Supporting information




**Supporting File**: anie72830‐sup‐0001‐SuppMat.docx.
The authors have cited additional references within the Supporting Information [99, 100, 101, 102, 103].

## Data Availability

The data that supports the findings of this study are available in the supplementary material of this article
