# Supplementary material for: Catalyst Hide‐and‐Seek Beneath Porous Support Surfaces: Pinpointing Active Site Distribution Through Resonance Energy Transfer
Source: Angew Chem Int Ed Engl. 2026 May 23;65(30):e4042285. doi: 10.1002/anie.4042285 (PMC13383038; doi:10.1002/anie.4042285)
Supplement: Supplementary file 1 — Supporting File: anie72830‐sup‐0001‐SuppMat.docx. The authors have cited additional references within the Supporting Information [99, 100, 101, 102, 103]. [file ANIE-65-e4042285-s001.docx]

Supporting Information
©Wiley-VCH 2021
69451 Weinheim, Germany

Catalyst Hide-and-Seek Beneath Porous Support Surfaces: Pinpointing Active Site Distribution through Resonance Energy Transfer

B. K. P. Maldeni Kankanamalage,^[a]^ W. J. Thompson,^[b]^ D. N. Smith,^[a]^ G. C. Thaggard,^[a]^ N. Wijerathne,^[c]^ I. Incognito,^[a]^ J. A. Byers,^†^*^[b]^ J. Niu,*^[b]^ and N. B. Shustova*^[a]^

**Abstract:**

Merging the high selectivity and efficiency of homogeneous catalysts with the recyclability of heterogeneous systems represents an attractive, industry-driven concept that can be realized through the “heterogenization” of existing molecular catalysts by incorporating them into porous solid-state matrices. The concept proposed herein uses Förster resonance energy transfer analysis to establish the first direct correlations among matrix topology, catalyst integration strategy, and active-site positioning in porous materials without employing fluorescent model systems. This catalyst mapping method can be applied to several classes of porous materials, including metal-organic frameworks and mesoporous silica. It addresses the existing challenges in relating the factors that control the spatial surface (re)distribution of molecular catalysts within such matrices before and after catalytic transformations. On the example of a series of six different catalyst-integrated materials, Å-level mapping of active site distribution was correlated with the nature of the porous host and the catalyst integration mechanism, which dictates the loading and accessibility of integrated catalysts. Thus, these studies provide a foundation for developing a framework to guide the design of recyclable heterogeneous catalysts with well-defined active-site distributions, both before and after catalytic transformations, which are key fundamental parameters for heterogeneous catalysis.

DOI: 10.1002/anie.2026XXXXX

Table of Contents

1. Materials and general considerations **S4**
2. Preparation of UiO-66 **S5**
3. Preparation of UiO-67  **S5**
4. Preparation of RuPNP@UiO-66 **S5**
5. Preparation of HG2@UiO-66 **S6**
6. Preparation of HG2@UiO-67 **S6**
7. Preparation of HG2@SBA-15 **S6**
8. Preparation of UiO-67-Ru(bpy)_3_-*pse* **S7**
9. Preparation of UiO-67-Ru(bpy)_3_-*dn* **S**7
10. Procedure for ring-closing metathesis reactions using HG2@UiO-67 **S7**
11. Procedure for ring-opening metathesis polymerization reactions using HG2@SBA-15 **S8**
12. Procedure for photooxidation of phenylboronic acid using UiO-67-Ru(bpy)_3_-*pse*/*dn* **S8**
13. ICP-OES analysis of catalyst-integrated UiO-66, UiO-67, and SBA-15 **S8**
14. Photoluminescence spectroscopy **S9**
15. **Table S1.** Summary of samples and conditions used in photoluminescence

quenching experiments **S9**

1. UV-vis spectroscopic and Förster resonance energy transfer (FRET) analysis **S9**
2. Other physical measurements **S10**
3. **Figure S1.** UV-vis absorbance and emission spectra of PVK in THF **S11**
4. **Figure S2.** UV-vis absorbance and emission spectra of PVK in DCM **S11**
5. **Figure S3.** UV-vis absorbance and emission spectra of PVK in pentane/toluene **S11**
6. **Figure S4.** UV-vis absorbance and emission spectra of PVK in DMF **S12**
7. **Figure S5.** Molar extinction coefficient spectra of RuPNP, benzene-1,4-dicarboxylic

acid, and ZrCl_4_ collected in THF **S12**

1. **Figure S6.** Molar extinction coefficient spectra of HG2, benzene-1,4-dicarboxylic acid,

and ZrCl_4_ collected in DCM **S12**

1. **Figure S7.** Molar extinction coefficient spectra of HG2, biphenyl-4,4-dicarboxylic

acid, and ZrCl_4_ collected in DCM **S13**

1. **Figure S8.** Molar extinction coefficient spectra of HG2 collected in pentane/toluene **S13**
2. **Figure S9.** Molar extinction coefficient spectra of [Ru(bpy)_2_(dcbpy)]Cl_2_, biphenyl-4,4-dicarboxylic acid, and ZrCl_4_ collected in DMF **S13**
3. **Figure S10.** Diffuse reflectance spectrum of RuPNP **S14**
4. **Figure S11.** Diffuse reflectance spectrum of HG2 **S14**
5. **Figure S12.** Diffuse reflectance spectrum of [Ru(bpy)_2_(dcbpy)]Cl_2_ **S14**
6. **Figure S13.** Diffuse reflectance spectra of UiO-66, UiO-67, and SBA-15 **S15**
7. **Table S2.** Photoluminescence quenching of PVK in the presence of

RuPNP@UiO-66 as a function of acceptor-to-donor molar ratio **S15**

1. **Figure S14.** Emission spectra of PVK in the presence and absence of UiO-66

collected in THF **S16**

1. **Figure S15.** Emission spectra of PVK, RuPNP@UiO-66, and UiO-66 collected in THF **S16**
2. **Table S3.** Photoluminescence quenching of PVK in the presence of

HG2@UiO-66 as a function of acceptor-to-donor molar ratio **S16**

1. **Figure S16.** Emission spectra of PVK in the presence and absence of UiO-66

collected in DCM **S17**

1. **Figure S17.** Emission spectra of PVK, HG2@UiO-66, and UiO-66 in DCM **S17**
2. **Table S4.** Photoluminescence quenching of PVK in the presence of

HG2@UiO-67 as a function of acceptor-to-donor molar ratio **S18**

1. **Figure S18.** Diffuse reflectance spectrum of UiO-67 overlaid with emission spectra

of PVK in the presence and absence of UiO-67 collected in DCM **S18**

1. **Figure S19.** Emission spectra of PVK, HG2@UiO-67, and UiO-67 in DCM **S19**
2. **Table S5.** Photoluminescence quenching of PVK in the presence of HG2@SBA-15

as a function of acceptor-to-donor molar ratio **S19**

1. **Figure S20.** Emission spectra of PVK in the presence and absence of SBA-15

collected in a pentane/toluene mixture **S20**

1. **Figure S21.** Emission spectra of PVK, HG2@SBA-15, and SBA-15 in a pentane/toluene

mixture **S20**

1. **Table S6.** Photoluminescence quenching of PVK in the presence of

UiO-67-Ru(bpy)_3_-*pse* as a function of acceptor-to-donor molar ratio **S21**

1. **Figure S22.** Emission spectra of PVK in the presence and absence of UiO-67

collected in DMF **S21**

1. **Figure S23.** Emission spectra of PVK, UiO-67-Ru(bpy)_3_-*pse*, and UiO-67 in DMF **S22**
2. **Table S7.** Photoluminescence quenching of PVK in the presence of

Ru(bpy)_3_-UiO-67-*dn* as a function of acceptor-to-donor molar ratio **S22**

1. **Figure S24.** Emission spectra of PVK in the presence and absence of UiO-67 in DMF **S23**
2. **Figure S25.** Emission spectra of PVK, UiO-67-Ru(bpy)_3_-*dn*, and UiO-67 in DMF **S23**
3. **Table S8.** Summary of FRET measurements **S24**
4. **Figure S26.** PXRD patterns of RuPNP@UiO-66 **S24**
5. **Figure S27.** PXRD patterns of HG2@UiO-66 **S25**
6. **Figure S28.** PXRD patterns of HG2@UiO-67 **S25**
7. **Figure S29.** PXRD patterns of UiO-67-Ru(bpy)_3_-*pse* **S25**
8. **Figure S30.** PXRD patterns of UiO-67-Ru(bpy)_3_-*dn* **S26**
9. **Figure S31.** ^1^H NMR spectrum of the product (2,5-dihydrofuran) of the RCM reaction

using HG2@UiO-67 **S26**

1. **Figure S32.** ^1^H NMR spectrum of the product (poly(cyclooctene)) of the ROMP reaction

using HG2@SBA-15 **S26**

1. **Figure S33.** ^1^H NMR spectrum of the product (phenol) of the photooxidation of

phenylboronic acid using UiO-67-Ru(bpy)_3_-*pse* **S27**

1. **Figure S34.** ^1^H NMR spectrum of the product (phenol) of the photooxidation of

phenylboronic acid using UiO-67-Ru(bpy)_3_-*dn* **S27**

1. **Figure S35.** Scanning electron micrographs of catalyst@MOF/SBA-15 samples **S27**
2. **Figure S36.** Scanning electron micrographs of UiO-67-Ru(bpy)_3_-*pse*/*dn* before and after

its use in the photooxidation of phenylboronic acid **S28**

1. **Figure S37.** Confocal microscopy image of UiO-67-Ru(bpy)_3_-*pse* **S28**
2. **References** **S28**

**Materials and general considerations**

Unless otherwise stated, all manipulations were carried out in the air using standard analytical procedures. Experiments carried out in an air-free environment were conducted under a positive pressure of N_2_ using standard glovebox or Schlenk line techniques.^[1]^

Zirconium (IV) chloride (99.5%, reactor grade, Alfa-Aesar), *cis*-bis(2,2'-bipyridine)dichlororuthenium(II) hydrate (97%, Fisher Chemical), benzene-1,4-dicarboxylic acid (99%, Sigma Aldrich), biphenyl-4,4-dicarboxylic acid (97.0%, TCI), 2,2'-bipyridine-4,4'-dicarboxylic acid (98%, Sigma Aldrich), (2,2’-bipyridine)-5,5’-dicarboxylic acid (98%, Sigma Aldrich), Santa Barbara Amorphous-15 (>99%, ACS Materials), Hoveyda–Grubbs second-generation catalyst (98%, Ambeed), triethylamine (99.0%, Sigma Aldrich), diethyl ether (≥99%, Fisher Chemical), allyl ether (98.0%, TCI), *N,N*-diisopropylethylamine (≥99.5%, Sigma Aldrich), acetic acid (99.7%, Optima LC/MS, Fisher Chemical), hydrofluoric acid (48–51% solution in water, Acros), hydrochloric acid (Certified ACS Plus, Fisher), anhydrous methanol (99.8%, Sigma Aldrich), ethanol (≥99.5%, Sigma Aldrich), acetonitrile (>99.5, Fisher Chemical), dichloromethane (99.9%, Fisher Chemical), *N*,*N*-dimethylformamide (Certified ACS, Fisher Chemical), pentane (>99%, HPLC, Fisher Chemical), toluene (>99%, HPLC, Fisher Chemical), tetrahydrofuran (Optima, Fisher Chemical), dichloromethane-*d*_2_ (99.8%, Cambridge Isotope Laboratories, Inc.), dimethyl sulfoxide-*d*_6_ (99.9%, Cambridge Isotope Laboratories, Inc.), and chloroform-*d* (>99%, Cambridge Isotope Laboratories, Inc.) were used as received. The compound 1,8-diazabicyclo[5.4.0]undec-7-ene (99%, Oakwood Chemical) was stirred over calcium hydride (90–95%, Alfa Aesar) for at least 24 hours before being vacuum-distilled and stored under nitrogen.

The compounds, 2,6-bis((di-tert-butyl-phosphino)methyl)pyridine (^tBu^PNP),^[2]^ (^tBu^PNP)Ru(CO)HCl (RuPNP),^[3]^ and bis(2,2’-bipyridine)(5,5’-dicarboxy-2,2’-bipyridine)ruthenium(II) chloride ([Ru(bpy)_2_(dcbpy)]Cl_2_)^[4]^ were synthesized based on modified literature procedures.

**Preparation of UiO-66**

Preparation of UiO-66 was carried out based on a reported literature procedure.^[5,6]^ In a 350-mL glass pressure vessel, ZrCl_4_ (373 mg, 1.60 mmol), benzene-1,4-dicarboxylic acid (266 mg, 1.60 mmol), acetic acid (18.0 mL), and *N*,*N*-dimethylformamide (DMF, 82.0 mL) were added. The vessel was sealed, and the reaction mixture was sonicated for 15 minutes. The resulting solution was then heated at 120 °C for 24 hours. Subsequently, the reaction mixture was cooled to room temperature. The solid was collected by centrifugation and then triturated with DMF (3 × 15 mL) by sonicating, centrifuging, and decanting. The solid was then triturated with methanol (3 × 15 mL) by sonicating, centrifuging, and decanting. After these procedures, the product was collected by centrifugation and dried overnight under vacuum, followed by additional drying at 70 °C in a vacuum chamber overnight. The resulting UiO-66 powder was collected in 79–90% yield (0.350–0.400 g, 0.210–0.240 mmol). The collected powder X-ray diffraction (PXRD) patterns matched the simulated pattern, and the crystallinity of the samples was maintained throughout postsynthetic integration of catalysts, exposure to catalytic reaction conditions, and photophysical studies based on PXRD analysis, as shown in Figure S26.

**Preparation of UiO-67**

The framework, UiO-67 was prepared based on a modified literature procedure.^[7]^ In a 350-mL glass pressure vessel, ZrCl_4_ (186 mg, 0.798 mmol), biphenyl-4,4-dicarboxylic acid (194 mg, 0.801 mmol), and DMF (100 mL) were added. To the vessel was then added acetic acid (12.4 mL) and triethylamine (1.20 mL). The vessel was then sealed, and the mixture was sonicated for ten minutes. The resulting solution was heated in an oven at 120 °C for 24 hours. Subsequently, the reaction mixture was cooled to room temperature. The solid was collected by centrifugation and then triturated with DMF (3 × 15 mL) by sonicating, centrifuging, and decanting. The solid was then triturated with methanol (3 × 15 mL) by sonicating, centrifuging, and decanting. The product was finally collected by centrifugation and dried overnight under vacuum, followed by additional drying at 110 °C in a vacuum chamber overnight. The resulting UiO-67 powder was isolated in 75% yield (212 mg, 0.100 mmol). The collected PXRD pattern matched the simulated pattern, and the crystallinity of the UiO-67 powder was maintained throughout postsynthetic integration of catalysts, exposure to catalytic reaction conditions, and photophysical studies based on PXRD analysis, as shown in Figures S28, S29, and S35.

**Preparation of RuPNP@UiO-66**

The RuPNP@UiO-66 MOF was prepared based on a modified literature procedure for aperture-opening encapsulation of catalysts in Zr-based MOFs.^[3]^ In a 20-mL crimp-seal vial equipped with a stir bar, UiO-66 (0.200 g, 120.0 µmol), (^tBu^PNP)Ru(CO)HCl (RuPNP; 10.0 mg, 17.8 µmol), and anhydrous methanol (10.0 mL) were combined. The vial was sealed, and then the reaction mixture was sparged with nitrogen for ten minutes. The reaction mixture was then sonicated for five minutes before being stirred and heated at 55 °C for 24 hours. Subsequently, the reaction mixture was cooled down to room temperature and transferred to a 20-mL vial. The prepared RuPNP@UiO-66 was collected by centrifugation and triturated with methanol (3 × 10 mL) by sonicating, centrifuging, and decanting. The product was then dried overnight in a vacuum chamber, affording a white solid (188 mg, 94%).

Catalyst-integrated RuPNP@UiO-66 was then subjected to the following pretreatment conditions prior to its use in catalytic experiments. In a 20-mL ampule, RuPNP@UiO-66 (0.100 g) was suspended in degassed DMF (15.0 mL) and 1,8-diazabicyclo[5.4.0]undec-7-ene (2.50 mL). The resulting mixture was then placed in a 450-mL stainless steel Parr reactor which was then sealed. The reaction vessel was purged for five minutes with carbon dioxide, pressurized to three bar, and then pressurized with hydrogen gas to 40 bar. The mixture was then heated to 130 °C for 45 minutes and subsequently cooled down to room temperature using a water bath. The pressure was slowly released, and then the reaction mixture was transferred into a 20-mL vial. The solid was isolated by centrifugation and then triturated with methanol (2 × 10 mL) by sonicating, centrifuging, and decanting. The white powder product was collected and dried overnight in a vacuum chamber to remove any residual solvents. The described encapsulation and pretreatment procedure was then repeated to yield sequentially encapsulated RuPNP@UiO-66.

The crystallinity of the framework RuPNP@UiO-66 was preserved after catalyst integration, as confirmed by PXRD analysis (Figures S26 and S35). Catalyst loading in UiO-66 was determined by inductively coupled plasma optical emission spectroscopy (ICP-OES), resulting in a loading of 0.005 wt%.

**Preparation of HG2@UiO-66**

The material HG2@UiO-66 was prepared based on a modified literature procedure in an air-free environment in a nitrogen-filled glovebox.^[7]^ In a 20-mL septum cap vial equipped with a stir bar, UiO-66 (0.350 g, 210 µmol), the Hoveyda-Grubbs second-generation catalyst (HG2; 35.0 mg, 5.60 µmol), and anhydrous acetonitrile (10.5 mL) were added. The vessel was sealed, and the reaction mixture was stirred at room temperature for 72 hours. Subsequently, the vessel was removed from the glovebox, and the solid was collected by centrifugation and then triturated with dichloromethane (DCM) (8 × 10 mL) by sonicating, centrifuging, and removing the resulting supernatant by syringe using Schlenk line techniques. The resulting powder was collected and dried overnight under vacuum to remove any residual solvent, affording a yellow solid (0.336 g, 96%). The crystallinity of the framework HG2@UiO-66 was preserved after catalyst encapsulation, as confirmed by PXRD analysis (Figures S27 and S35). Catalyst loading in UiO-66 was determined by ICP-OES, resulting in a loading of 0.059 wt%.

**Preparation of HG2@UiO-67**

The material HG2@UiO-67 was prepared based on a modified literature procedure in an air-free environment in a nitrogen-filled glovebox.^[7]^ In a 20-mL septum cap vial equipped with a stir bar, UiO-67 (0.350 g, 165 µmol), HG2 (35.0 mg, 56.0 µmol), and anhydrous acetonitrile (10.5 mL) were added. The vessel was sealed, and the reaction mixture was stirred at room temperature for 72 hours. Subsequently, the vessel was removed from the glovebox, and the solid was collected by centrifuging and then triturated with DCM (8 × 10.0 mL) by sonicating, centrifuging, and removing the resulting supernatant by syringe using Schlenk line techniques. The resulting powder was collected and dried overnight in a vacuum chamber to remove any residual solvent, affording a yellow solid. The crystallinity of HG2@UiO-67 was preserved, as confirmed by PXRD analysis (Figures S28 and S35). Catalyst loading in UiO-67 was determined by ICP-OES, resulting in a loading of 0.101 wt%.

**Preparation of HG2@SBA-15**

The material HG2@SBA-15 was prepared based on a modified literature procedure in an air-free environment in a nitrogen-filled glovebox.^[8]^ In a 20-mL septum cap vial equipped with a stir bar, Santa Barbara Amorphous-15 (SBA-15; 0.400 g, 6.66 mmol), HG2 (8.40 mg, 13.4 µmol), and DCM (12.0 mL) were added. The vessel was sealed, and the reaction mixture was vigorously stirred at room temperature for four hours. Subsequently, the solvent was evaporated under reduced pressure, and the resulting yellow-green powder was collected and dried under vacuum overnight, affording a yellow-green solid (0.380 g, 95%, Figure S35). Catalyst loading in SBA-15 was determined by ICP-OES, resulting in a loading of 0.155 wt%.

**Preparation of** **UiO-67-Ru(bpy)_3_-*pse***

The material UiO-67-Ru(bpy)_3_-*pse* was prepared based on a modified literature procedure for postsynthetic encapsulation of molecular catalysts in MOFs.^[9]^ In a 10-dram vial equipped with a stir bar, UiO-67 (0.250 g, 118 µmol), [Ru(bpy)_2_(dcbpy)]Cl_2_ (bis(2,2’-bipyridine)(5,5’-dicarboxy-2,2’-bipyridine)ruthenium(II) chloride) (15.0 mg, 20.6 µmol), ethanol (18.8 mL), and deionized water (6.25 mL) were added. The vessel was sealed, and the reaction mixture was sonicated for 15 minutes before being stirred and heated at 85 °C for 24 hours. Subsequently, the reaction mixture was cooled to room temperature. The reaction mixture was then transferred to a 50-mL vial and triturated with methanol (3 × 30 mL) by sonicating, centrifuging, and decanting. This process was then repeated with deionized water (30.0 mL) and ethanol (2 × 30 mL). The resulting orange solid was then suspended in ethanol (30.0 mL) for three days, and the solvent was exchanged daily. The orange powder product was then dried in a vacuum chamber overnight at room temperature followed by drying at 55 °C for three hours under vacuum, affording an orange solid (0.240 g, 96%). The crystallinity of UiO-67-Ru(bpy)_3_-*pse* was preserved after catalyst encapsulation, as confirmed by PXRD analysis (Figures S29 and S36). Catalyst loading in UiO-67 was determined by ICP-OES, resulting in a loading of 0.161 wt%.

**Preparation of UiO-67-Ru(bpy)_3_-*dn***

The material UiO-67-Ru(bpy)_3_-*dn*, was prepared based on a modified literature procedure.^[4]^ In a 100-mL glass pressure vessel, ZrCl_4_ (181 mg, 0.778 mmol), biphenyl-4,4-dicarboxylic acid (172 mg, 0.708 mmol), 2,2'-bipyridine-4,4'-dicarboxylic acid (11.0 mg, 15.6 µmol), [Ru(bpy)_2_(dcbpy)]Cl_2_ (17.0 mg, 23.3 µmol), DMF (26.3 mL), and acetic acid (1.44 mL) were added. The vessel was sealed, and then the reaction mixture was sonicated for ten minutes. The resulting solution was then heated at 120 °C for 24 hours. Subsequently, the reaction mixture was cooled to room temperature. The solid was collected by centrifugation and then triturated with DMF (3 × 30 mL) by sonicating, centrifuging, and decanting. The solid was then triturated with methanol (3 × 30 mL) by sonicating, centrifuging, and decanting. The resulting orange solid was then suspended in ethanol (30.0 mL) for three days, and the solvent was exchanged daily. The orange powder product was then dried under vacuum overnight at room temperature, followed by drying for three hours under vacuum at 55 °C affording an orange solid (0.245 mg, 98%). The crystallinity of UiO-67-Ru(bpy)_3_-*dn* was preserved, as confirmed by PXRD analysis (Figures S30 and S36). Catalyst loading in UiO-67 was determined by ICP-OES, resulting in a loading of 0.388 wt%.

**Ring-closing metathesis reactions using HG2@UiO-67**

The material HG2@UiO-67 was exposed to ring-closing metathesis (RCM) conditions based on a modified literature procedure in a nitrogen-filled glovebox.^[7]^ In a 3-mL vial equipped with a stir bar, HG2@UiO-67 (120 mg, 0.101 wt%), dichloromethane-*d*_2_ (0.830 mL), and allyl ether (8.70 µL, 71.2 µmol) were added. The vessel was sealed, and the reaction mixture was vortexed briefly before stirring at room temperature for one hour. Subsequently, the reaction was removed from the glovebox, quenched with ethyl vinyl ether and diluted with CD_2_Cl_2_. The solid was collected by centrifugation, and the resulting white solid was then dried overnight in a vacuum chamber. The reaction conversion was determined by ^1^H nuclear magnetic resonance (NMR) spectroscopy of the supernatant (Figure S31). The crystallinity of HG2@UiO-67 was preserved as confirmed by PXRD analysis (Figure S28). Catalyst loading in UiO-67 after exposure to ring-closing metathesis reaction conditions was determined by ICP-OES, resulting in 0.084 wt% loading.

**Ring-opening metathesis polymerization using HG2@SBA-15**

The material HG2@SBA-15 was exposed to ring-opening metathesis polymerization (ROMP) conditions based on a modified literature procedure in a nitrogen-filled glovebox.^[10]^ In a 5-mL Schlenk tube, HG2@SBA-15 (150 mg, 0.155 wt%), pentane (2.20 mL), and cyclooctene (188 µL) were added. The vessel was sealed, removed from the glovebox, and the reaction mixture was heated at 30 °C for three hours. Subsequently, the reaction was opened to atmosphere and quenched with ethyl vinyl ether and diluted with chloroform-*d*. The solid was collected by centrifugation, and the resulting white powder was then dried overnight in a vacuum chamber. The reaction conversion was determined using ^1^H NMR spectroscopy of the supernatant (Figure S32). Catalyst loading in SBA-15 after exposure to the ROMP reaction conditions was determined by ICP-OES, resulting in a loading of 0.104 wt%.

**Photooxidation of phenylboronic acid using UiO-67-Ru(bpy)_3_-*pse*/*dn***

The materials UiO-67-Ru(bpy)_3_-*pse* and UiO-67-Ru(bpy)_3_-*dn* were exposed to phenylboronic acid photooxidation reaction conditions based on a modified literature procedure.^[9]^ In a 10-mL round-bottom flask, UiO-67-Ru(bpy)_3_-*pse*/*dn* (1.0 equiv. Ru), phenylboronic acid, pinacol ester (100 equiv.), DMF (100 µM Ru), and *N*,*N*-diisopropylethylamine (120 equiv.) were added. The reaction mixture was sonicated for ten minutes and then irradiated with UV light (370 nm) for 24 hours at room temperature. Subsequently, the reaction mixture was centrifuged and then triturated with methanol (10 mL) by sonicating, centrifuging, and decanting. The resulting solid was then dried overnight in a vacuum chamber. The reaction conversion was determined by ^1^H NMR spectroscopy of the concentrated supernatant in chloroform-*d* (Figures S33 and S34). Catalyst loading in UiO-67-Ru(bpy)_3_-*pse* and UiO-67-Ru(bpy)_3_-*dn* after catalysis was determined by ICP-OES, resulting in loadings of 0.168 wt% and 0.268 wt%, respectively.

**ICP-OES analysis of catalyst-integrated UiO-66, UiO-67, and SBA-15**

Prior to ICP-OES analysis of catalyst-integrated samples, four standard solutions were prepared by dilution from commercially available zirconium (999 ± 5 µg/mL), ruthenium (999 ± 5 µg/mL), and phosphorus (1000 ± 10 µg/mL) standards using serial dilution in grade A volumetric glassware to cover the expected concentration ranges. The standards were then employed in a calibration curve to determine the loading of the catalyst in a tested solid. These standards consisted of Zr/Ru/P concentrations in µg/mL at the proportions: 250/5/5, 100/2/2, 25/0.5/0.5, and 2.5/0.05/0.05.

Solid RuPNP@UiO-66, HG2@UiO-66, and UiO-67-Ru(bpy)_3_-*pse*/*dn* materials (approximately 10.0 mg, weighed exactly) were then weighed into a 1.5-mL Teflon vial before DMSO (0.500 mL) and two drops of 15 wt% aqueous hydrofluoric acid solution were added in sequence. The mixture was sonicated for three minutes and left to digest at room temperature for three hours. The digested samples were then heated to approximately 130 °C overnight in a sand bath open to the air to remove solvent. The resulting solid was then dissolved in a 20-mL glass scintillation vial using a mixture (10% v/v) of hydrochloric acid in deionized water (0.500 mL). Each sample was diluted with additional deionized water (4.50 mL). Finally, an aliquot (0.500 mL) of each sample was further diluted with deionized water (4.50 mL). Both prepared solutions were then analyzed by ICP-OES.

The material HG2@UiO-67 (approximately 2.0 mg) was transferred into a 20-mL glass scintillation vial. Concentrated hydrochloric acid (0.500 mL) was added, and the mixture was sonicated until the solid was fully dispersed for three minutes. Ultrapure water (2.00 mL, Milli-Q) was then added, and the suspension was sonicated for 30 minutes. The digested sample was diluted to 10.0 mL with additional ultrapure water using a 10-mL volumetric flask, filtered through a 0.22-µm PTFE syringe filter, and analyzed by ICP-OES. Solid HG2@SBA-15 sample (2.00–4.00 mg) was weighed into a 20-mL glass scintillation vial. Hydrofluoric acid (40 µL) was added, followed by freshly prepared aqua regia (~5.00 mL). The mixture was heated to boiling on a hot plate for one hour, cooled to room temperature, and diluted with 2% HNO_3_ to a final concentration of ~100 ppb for analysis by ICP-OES.

**Photoluminescence spectroscopy**

Steady-state photoluminescence spectra were acquired on an Edinburgh FS5 fluorescence spectrometer equipped with a 150 W Continuous Wave Xenon lamp source for excitation and a Molecular Devices spectrometer with a 50 W Xenon flash lamp. Emission measurements were performed using a 1.0-cm quartz cuvette.

Steady-state photoluminescence spectroscopy was used to analyze catalyst@MOF/SBA-15 samples to determine the photoluminescence quenching efficiency of the incorporated catalyst (acceptor; solid) and PVK (donor; solution) by varying the acceptor/donor molar ratio to identify the maximum quenching (Table S1). For this, emission spectra of PVK solutions (0.800–1.00 mL) at different concentrations were collected. After collecting the initial PVK emission spectra, catalyst@MOF/SBA-15 powders were added to a quartz cuvette containing PVK in tetrahydrofuran (THF), DCM, pentane/toluene, or DMF (see Table S1 for the specific experimental conditions). The emission spectra of the resulting suspensions were recorded (*λ*_ex_ = 310 nm or 345 nm) for varying acceptor/donor molar ratios. The quenching efficiency was determined by comparing the decrease in intensity at the donor’s emission maximum (*λ*_em(max)_ = 375 nm) after the addition of the acceptor solid sample (Figures 2–5 in the main text). An acceptor-to-donor molar ratio where the change in quenching efficiency as a function of acceptor concentration began to plateau (i.e., a maximum quenching efficiency), was selected for control emission measurements (Tables S1–S7 and Figures S14, S16, S18, S20, S22, and S24).

**Table S1.** Summary of samples and conditions used for photoluminescence quenching studies.

| **entry** | **system** | **PVK (donor) concentrations, µM** | **acceptor/ donor molar ratios** | **selected acceptor/donor molar ratio** | **solvent** | ***λ*_ex_, nm** |
| --- | --- | --- | --- | --- | --- | --- |
| 1 | RuPNP@UiO-66 | 0.200–6.06 | 0.10–3.0 | 0.15 | THF | 310 |
| 2 | HG2@UiO-66 | 2.51–30.46 | 0.09–1.15 | 0.19 | DCM | 345 |
| 3 | HG2@UiO-67 | 1.20–8.70 | 0.22–2.1 | 0.25 | DCM | 345 |
| 4 | HG2@SBA-15 | 3.97–198 | 0.076–3.8 | 0.11 | pentane/toluene (v/v, 9:1) | 310 |
| 5 | UiO-67-Ru(bpy)_3_-*pse* | 6.72–3.37 | 0.74–1.5 | 0.89 | DMF | 345 |
| 6 | UiO-67-Ru(bpy)_3_-*dn* | 2.52–5.61 | 2.7–6.0 | 3.6 | DMF | 345 |

**UV-vis spectroscopic and Förster resonance energy transfer (FRET) analysis**

As a first step toward FRET analysis of the prepared catalyst-integrated matrices, control experiments demonstrating that the host matrix does not significantly participate in energy transfer processes with the PVK donor were performed. For this, the molar extinction coefficients of the catalysts were compared to the components of the porous material (e.g., SBA-15 or metal salts and organic linkers) in solution and in the solid state.

For this, 0.250–1.00 mg of each catalyst (RuPNP, HG2, or [Ru(bpy)_2_(dcbpy)]Cl_2_) was used to prepare 20.0–325 µM stock solutions, followed by serial dilution to obtain concentrations of 90.0, 2.00, 29.0, 32.0, and 25 µM in the following solvents: THF, DCM, pentane/toluene (v/v, 9:1), and DMF. Additionally, 6.0–12 mg of either benzene-1,4-dicarboxylic acid or biphenyl-4,4-dicarboxylic acid (corresponding to UiO-66 or UiO-67, respectively) were suspended in 1.00–5.00 mL of THF, DCM, and DMF. After sonication and centrifugation to remove solid particles, the absorbance of each solution was measured in a 1.0-cm quartz cuvette. Similarly, 10.0 mg of ZrCl_4_ was suspended in 2.00 mL of the same solvents, and absorbance of the resulting suspension was measured. The resulting absorbance spectra showed that the molar extinction coefficients of the catalysts were significantly higher than those of the corresponding organic linkers and metal salt. This confirms that the energy transferred from the excited donor (PVK) is absorbed by the catalyst (acceptor) located in the MOF/SBA-15 during the FRET process rather than by the organic linkers and metal salts. The UV-vis absorbance spectra for all control experiments are given in Figures S5–S9. Additionally, the UV-vis absorbance and emission spectra of PVK (7.25 µM) were collected in same solvents (THF, DCM, pentane/toluene (v/v, 9:1), and DMF) and are shown in Figures S1–S4.

FRET analysis of the prepared catalyst-integrated porous materials was carried out by first calculating the spectral overlap function, *J*, from the experimental donor emission spectra (PVK; 4.04, 15.0, 8.09, 132, 5.61, and 4.23 µM in THF, DCM, pentane/toluene, and DMF), where the molecular weight of 23,000 Mn (determined by gel permeation chromatography) was used to determine the moles of PVK, and acceptor absorption spectra (RuPNP, HG2, [Ru(bpy)_2_(dcbpy)]Cl_2_; 90.0, 2.00, 29.0, 32.0 and 25 µM in THF, DCM, pentane/toluene (v/v, 9:1), and DMF, respectively) using the following equation.^[11]^

$$J=\int f(\lambda)d\lambda, f(\lambda) = F_{D}(\lambda)\varepsilon_{A}(\lambda)\lambda^{4} (\mathrm{Eq}. S1)$$

where *F*_D_(*λ*) is the donor emission spectrum normalized to the unit area and *ε*_A_(*λ*) is the molar extinction coefficient spectrum of the acceptor.^[10]^ The calculated overlap function was used for estimation of the corresponding Förster critical radius, *R*_o_, i.e., the distance at which energy transfer efficiency is 50%, by the following equation:

$$R_{o}(cm) = \left( 8.79\times{10}^{-25}\times\kappa^{2}n^{-4}Q_{d}J \right)^{\frac{1}{6}} (Eq.S2)$$

where *Q*_d_ = *k*_r_ × *τ*_D_ (*k*_r_ = donor radiative rate), *κ* is an orientation factor taken to be *κ*^2^ = 2/3 corresponding to randomized orientations, and *n* is the effective refractive index approximated to be 1.^[11]^ The function, *f*(*λ*), is plotted in Figures 2–5 in the main text.

**Other physical measurements**

PXRD patterns were collected on a Bruker D2 PHASER or Rigaku Miniflex 6G diffractometer at a scan rate of 6 °/minute with accelerating voltage and current of 40 kV and 15 mA, respectively. The confocal microscopy images were collected on a Leica Stellaris 8 STED/WLL spectral microscope equipped with a variable wavelength White Light Laser. The SEM images were collected on a JEOL JSM-6340F Scanning Electron Microscope. NMR spectra were recorded at ambient temperature on Varian VNMRS operating at 600 MHz for ^1^H NMR spectroscopy. ^1^H NMR spectra were referenced to the residual ^1^H peaks of deuterated solvents. Absorbance spectra were collected on an Evolution 350 or Molecular Devices SpectraMax M5 spectrometer. Samples were loaded in a 1.00-mL quartz cuvette and referenced to their respective pure solvents. ICP-OES analysis was performed on an Agilent 5100 VDV and Agilent 5800 inductively coupled plasma optical emission spectrometers. Diffuse reflectance spectra of MOF samples, SBA-15, and catalysts were collected using a ThermoFisher Evolution 350 UV-vis spectrometer paired with Harrick Scientific Praying Mantis Diffuse Reflection accessory. The samples were prepared by mixing 1–2 mg of UiO-66/UiO-67/SBA-15/catalysts with powdered BaSO_4_ (Figures S10–S13).


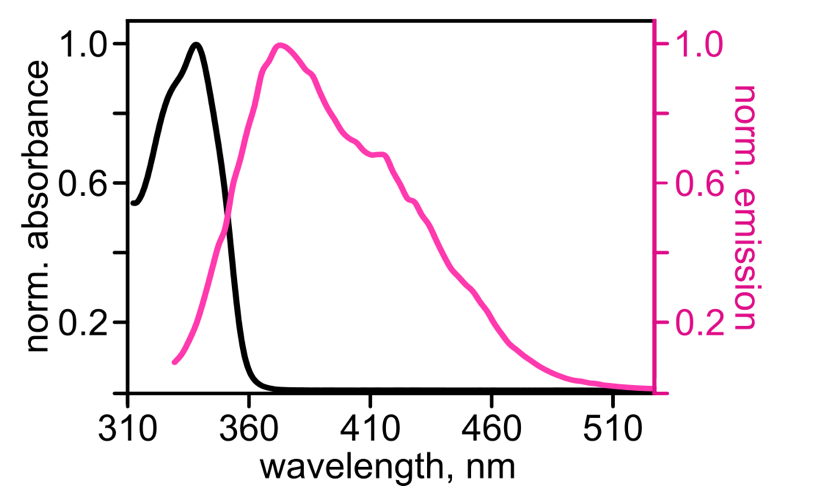


**Figure S1.** Normalized absorbance (black) and emission (pink) spectra of PVK (7.25 µM in THF, *λ*_ex_ = 310 nm).


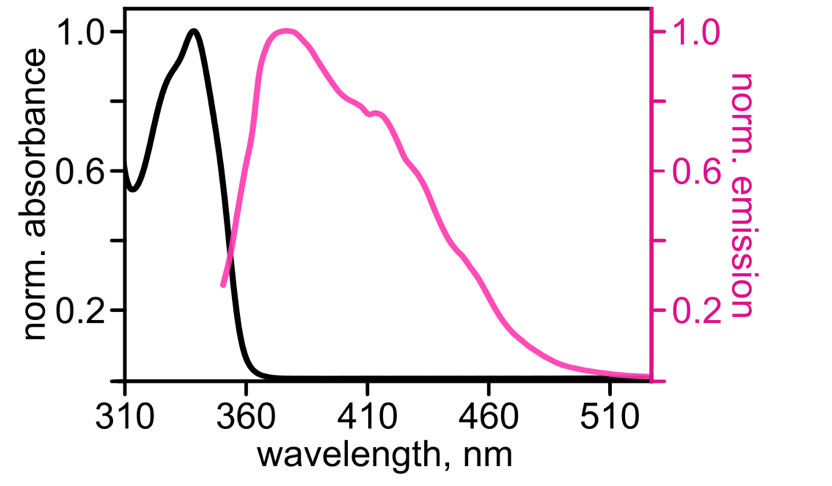


**Figure S2.** Normalized absorbance (black) and emission (pink) spectra of PVK (7.25 µM in DCM, *λ*_ex_ = 345 nm).


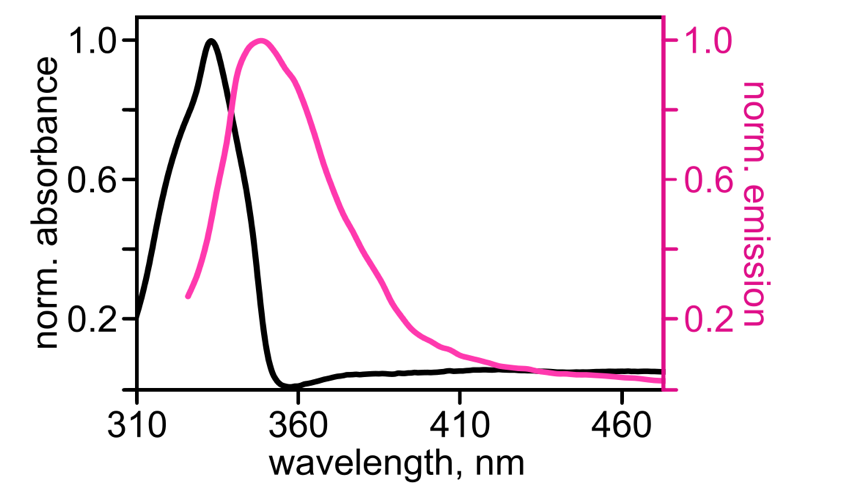


**Figure S3.** Normalized absorbance (black) and emission (pink) spectra of PVK (7.25 µM in pentane/toluene: v/v, 9:1, *λ*_ex_ = 310 nm).


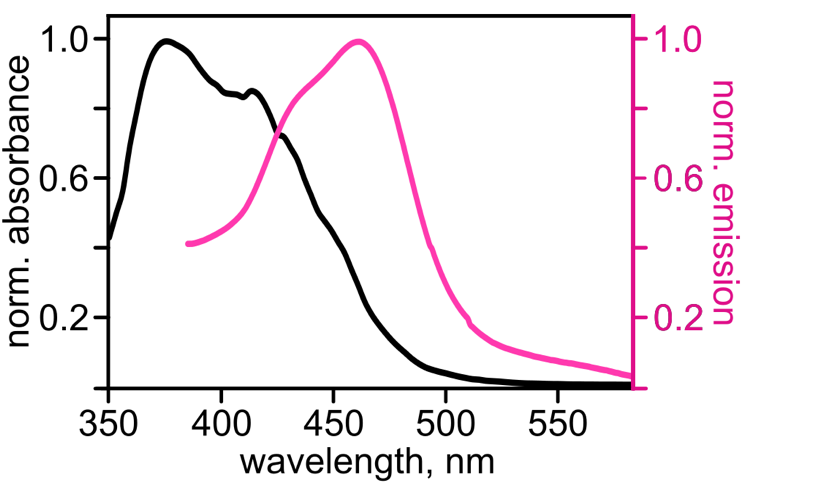


**Figure S4.** Normalized absorbance (black) and emission (pink) spectra of PVK (7.25 µM in DMF, *λ*_ex_ = 345 nm).

**
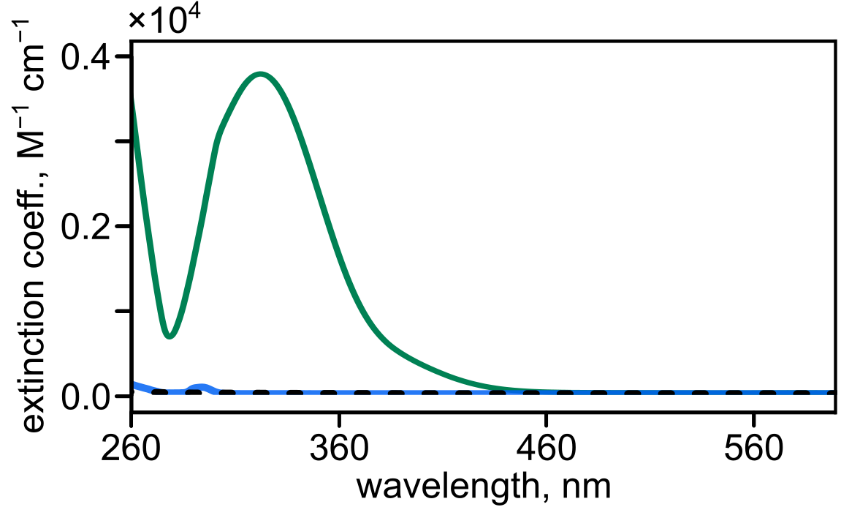
**

**Figure S5.** Molar extinction coefficient spectra of RuPNP (solid green line), benzene-1,4-dicarboxylic acid (solid blue line), and ZrCl_4_ (dashed black line) in THF.


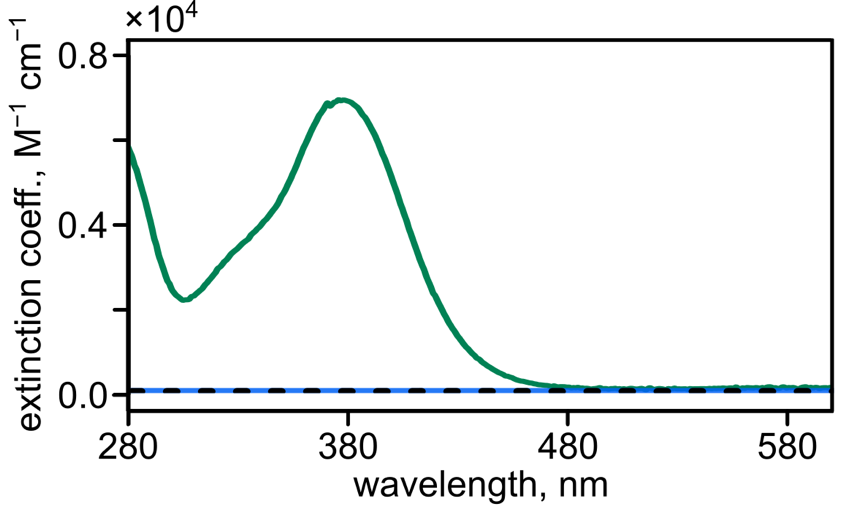


**Figure S6.** Molar extinction coefficient spectra of HG2 (solid green line), benzene-1,4-dicarboxylic acid (solid blue line), and ZrCl_4_ (dashed black line) in DCM.


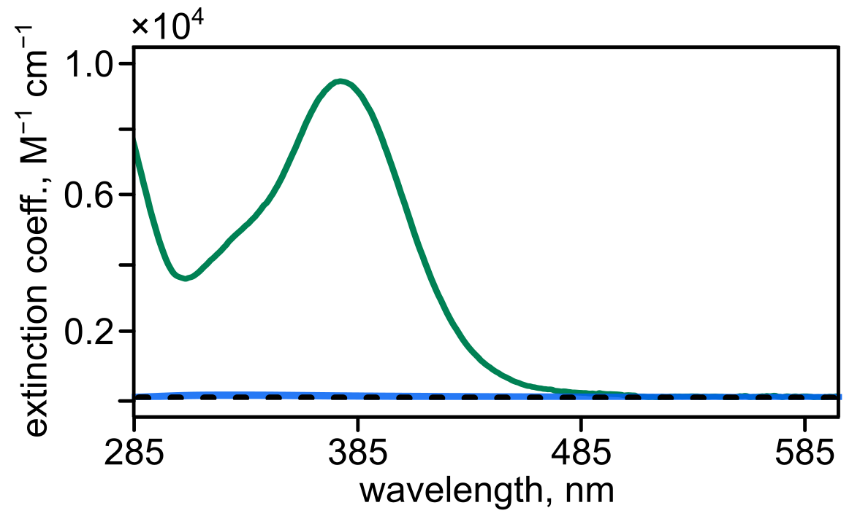


**Figure S7.** Molar extinction coefficient spectra of HG2 (solid green line), biphenyl-4,4-dicarboxylic acid (solid blue line), and ZrCl_4_ (dashed black line) in DCM.

**
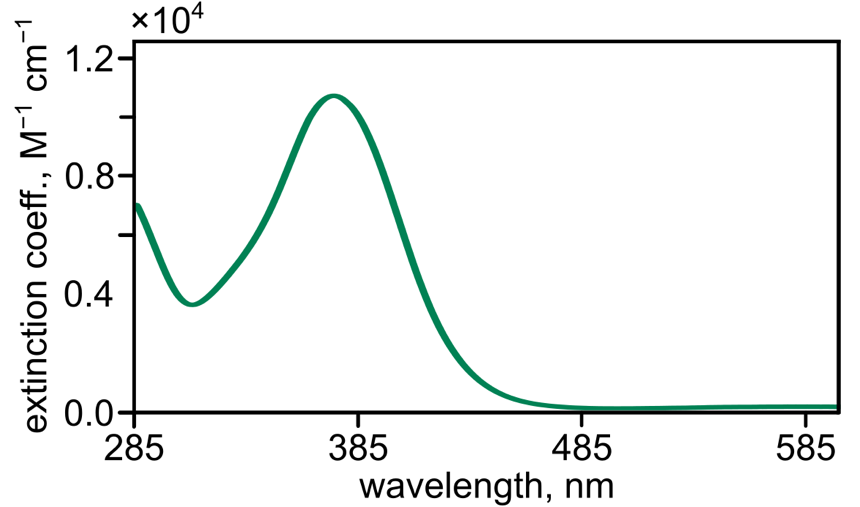
**

**Figure S8.** Molar extinction coefficient spectra of HG2 (solid green line) in a 9:1 v/v mixture of pentane and toluene.

**
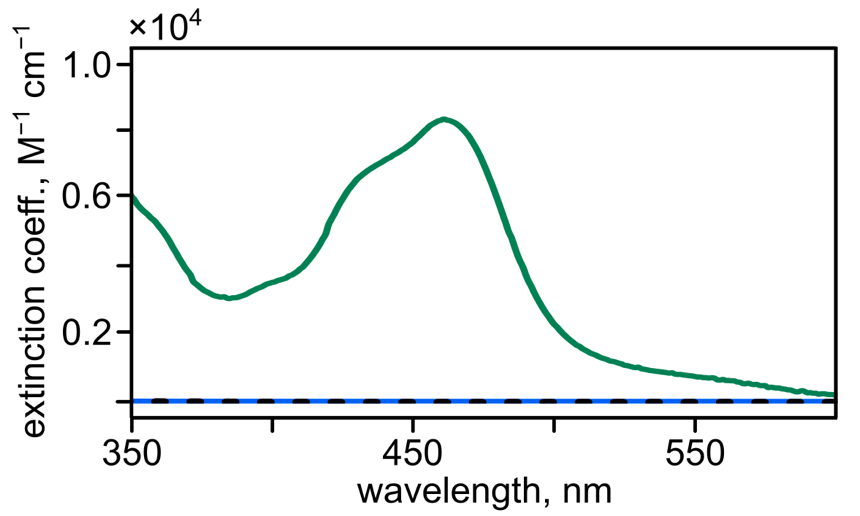
**

**Figure S9.** Molar extinction spectra of [Ru(bpy)_2_(dcbpy)]Cl_2_ (solid green line), biphenyl-4,4-dicarboxylic acid (solid blue line), and ZrCl_4_ (dashed black line) in DMF.


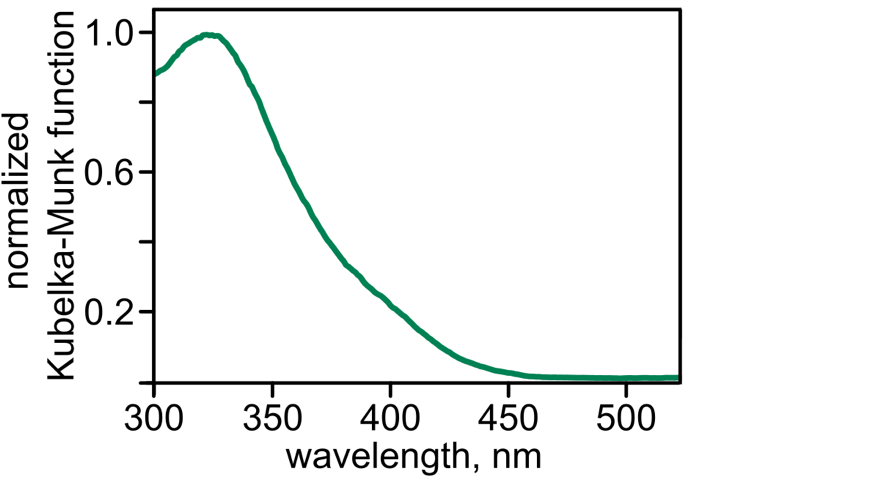


**Figure S10.** Normalized diffuse reflectance spectrum of RuPNP.

**
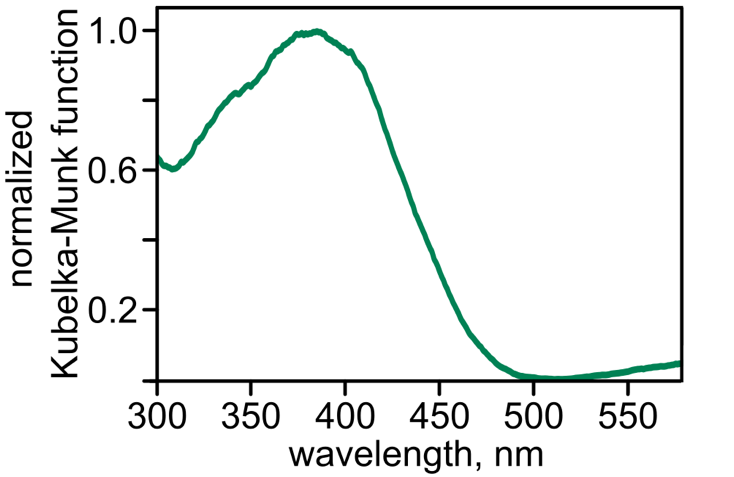
**

**Figure S11.** Normalized diffuse reflectance spectrum of HG2.

**
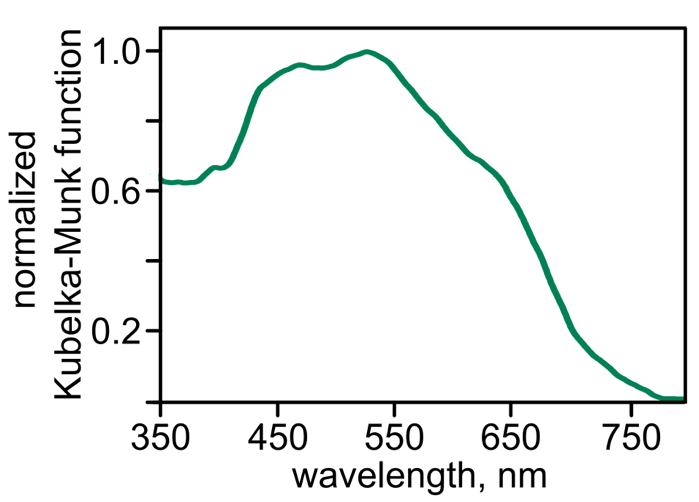
**

**Figure S12.** Normalized diffuse reflectance spectrum of [Ru(bpy)_2_(dcbpy)]Cl_2_.

**
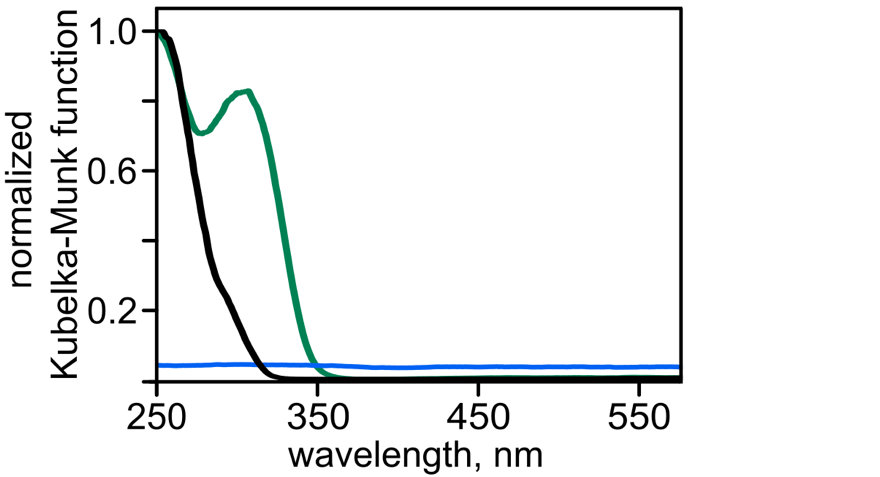
**

**Figure S13.** Diffuse reflectance spectra of UiO-66 (black), UiO-67 (green), and SBA-15 (blue).

**Table S2.** Summary of photoluminescence quenching of PVK in the presence of RuPNP@UiO-66 as a function of acceptor-to-donor molar ratio. Data were acquired from photoluminescence spectra collected in THF.

| **entry** | **acceptor/donor molar ratio** | ***E*, %** |
| --- | --- | --- |
| 1 | 0.10 | 25 ± 2 |
| 2 | 0.15 | 36 ± 1 |
| 2*^a^* | – | 0.4 ± 0.3 |
| 3 | 0.21 | 36 ± 2 |
| 4 | 3.0 | 36 ± 2 |

*^a^*the control experiment for the system in the absence of the acceptor

**
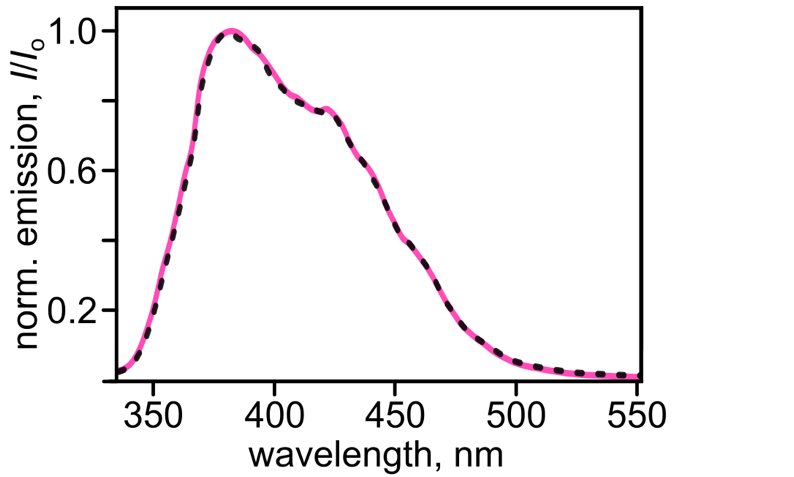
**

**Figure S14.** Emission spectra of PVK (solid pink line, *λ*_ex_ = 310 nm; 4.04 µM in THF) and PVK in a UiO-66 suspension (*λ*_ex_ = 310 nm; dashed black line) with an acceptor-to-donor molar ratio of 0.15.


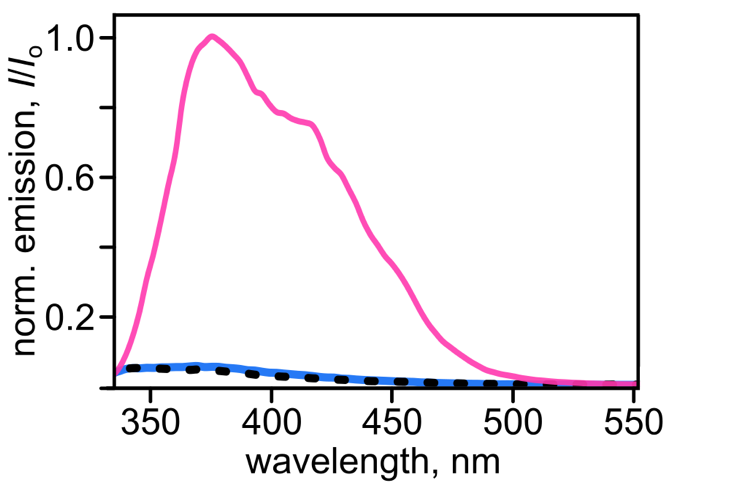


**Figure S15.** Emission spectra of PVK (solid pink line; *λ*_ex_ = 310 nm; 4.04 µM in THF), RuPNP@UiO-66, and UiO-66 (*λ*_ex_ = 310 nm, solid blue and dashed black lines, respectively).

**Table S3.** Summary of photoluminescence quenching of PVK in the presence of HG2@UiO-66 as a function of acceptor-to-donor molar ratio. Data were acquired from photoluminescence spectra collected in DCM.

| **entry** | **acceptor/donor molar ratio** | ***E*, %** |
| --- | --- | --- |
| 1 | 0.09 | 16 ± 1 |
| 2 | 0.19 | 23 ± 4 |
| 2*^a^* | – | 0.4 ± 0.3 |
| 3 | 0.24 | 22 ± 2 |
| 4 | 0.38 | 22 ± 2 |
| 5 | 1.15 | 22 ± 3 |

*^a^*the control experiment for the system in the absence of the acceptor


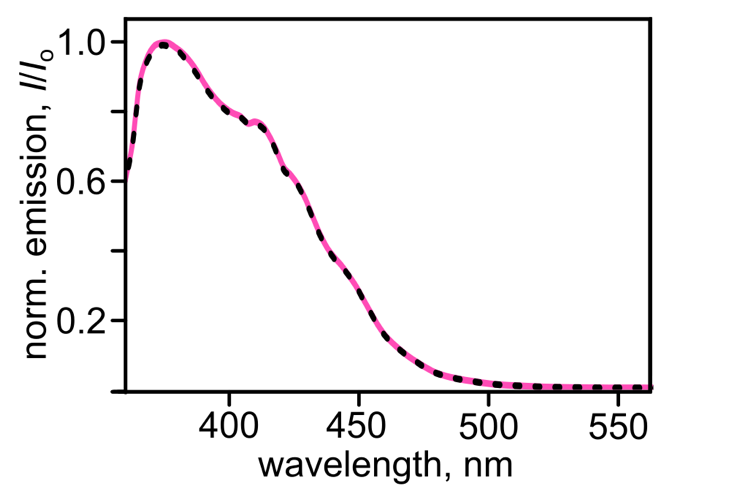


**Figure S16.** Emission spectra of PVK (solid pink line, *λ*_ex_ = 345 nm; 15.0 µM in DCM) and PVK in a UiO-66 suspension (*λ*_ex_ = 345 nm; dashed black line) with an acceptor-to-donor molar ratio of 0.19.

**
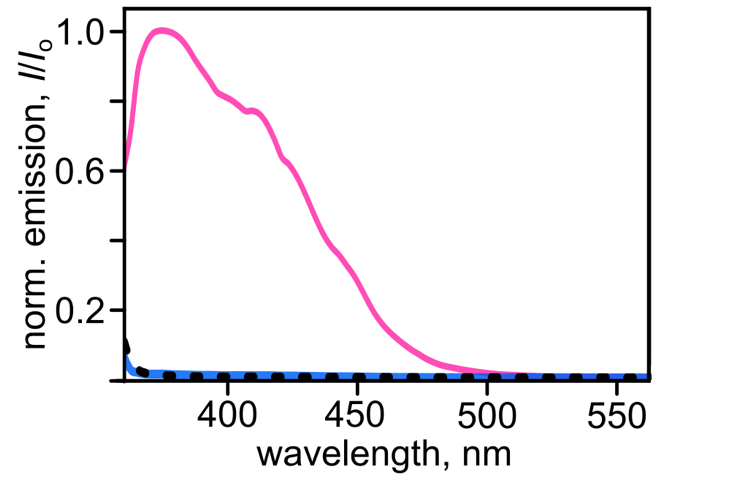
**

**Figure S17.** Emission spectra of PVK (solid pink line; *λ*_ex_ = 345 nm; 15.0 µM in DCM), HG2@UiO-66, and UiO-66 (*λ*_ex_ = 345 nm, solid blue and dashed black lines).

**Table S4.** Summary of photoluminescence quenching of PVK in the presence of HG2@UiO-67 as a function of acceptor-to-donor molar ratio. Data were acquired from photoluminescence spectra collected in DCM.

| **entry** | **acceptor/donor molar ratio** | ***E*, %** | ***E*_HG2@UiO-67_, %** |
| --- | --- | --- | --- |
| 1 | 0.22 | 17 ± 3 | 14 ± 3 |
| 1*^a^* | – | 3 ± 1 | – |
| 2 | 0.25 | 19 ± 3 | 14 ± 3 |
| 2*^a^* | – | 5 ± 1 | – |
| 2*^b^* | 0.25 | 18 ± 0.3 | 13 ± 3 |
| 3 | 0.40 | 19 ± 3 | 14 ± 3 |
| 3*^a^* | – | 5 ± 1 | – |
| 4 | 2.1 | 17 ± 2 | 13 ± 3 |
| 4*^a^* | – | 4 ± 2 | – |

*^a^*the control experiment for the system in the absence of the acceptor

*^b^* photoluminescence quenching of PVK by HG2@UiO-67 after its use in the RCM reaction


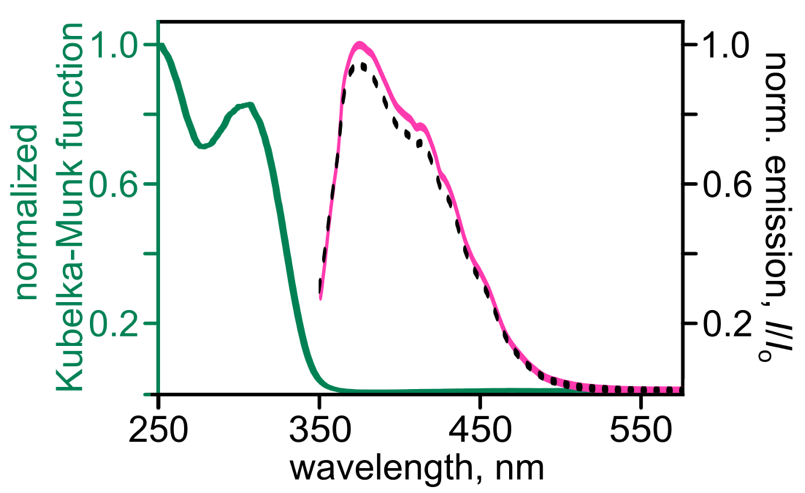


**Figure S18.** Diffuse reflectance spectrum of UiO-67 (solid green line) overlaid with emission spectra of PVK (solid pink line, *λ*_ex_ = 345 nm; 8.09 µM in DCM) and PVK in a UiO-67 suspension (*λ*_ex_ = 345 nm; dashed black line) with an acceptor-to-donor molar ratio of 0.25.


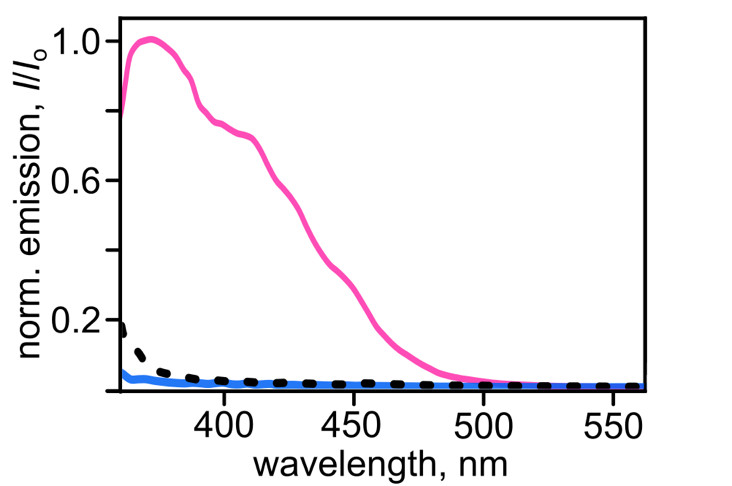


**Figure S19.** Emission spectra of PVK (solid pink line; *λ*_ex_ = 345 nm; 8.09 µM in DCM), HG2@UiO-67, and UiO-67 (*λ*_ex_ = 345 nm, solid blue and dashed black lines).

**Table S5.** Summary of photoluminescence quenching of PVK in the presence of HG2@SBA-15 as a function of acceptor-to-donor molar ratio. Data were acquired from photoluminescence spectra collected in 9:1 v/v mixture of pentane and toluene.

| **entry** | **acceptor/donor molar ratio** | ***E*, %** |
| --- | --- | --- |
| 1 | 0.076 | 10 ± 2 |
| 2 | 0.11 | 13 ± 1 |
| 2*^a^* | – | 1.2 ± 1.1 |
| 2*^b^* | 0.11 | 13 ± 2 |
| 3 | 0.15 | 13 ± 2 |
| 4 | 0.20 | 13 ± 2 |
| 5 | 3.8 | 13 ± 2 |

*^a^*the control experiment for the system in the absence of the acceptor

*^b^*photoluminescence quenching of PVK by HG2@SBA-15 after its use in the ROMP

reaction


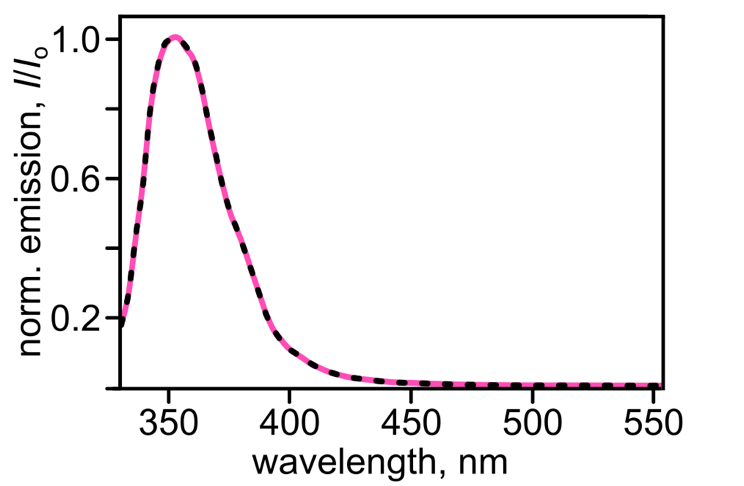


**Figure S20.** Emission spectra of PVK (solid pink line, *λ*_ex_ = 310 nm; 132 µM in a 9:1 v/v mixture of pentane and toluene) and PVK in an SBA-15 suspension (*λ*_ex_ = 310 nm; dashed black line) with an acceptor-to-donor molar ratio of 0.11.


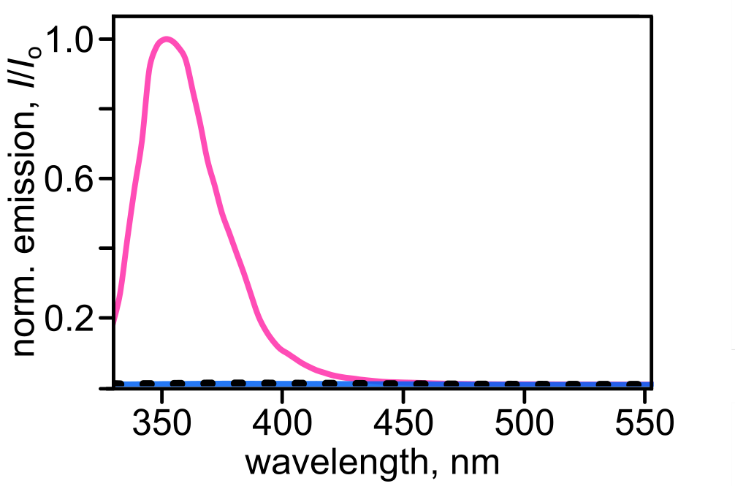


**Figure S21.** Emission spectra of PVK (solid pink line; *λ*_ex_ = 310 nm; 132 µM in a 9:1 v/v mixture of pentane and toluene), HG2@SBA-15, and SBA-15 (*λ*_ex_ = 310 nm, solid blue and dashed black lines).

**Table S6.** Summary of photoluminescence quenching of PVK with UiO-67-Ru(bpy)_3_-*pse* as a function of acceptor-to-donor molar ratio. Data were acquired from photoluminescence spectra collected in DMF.

| **entry** | **acceptor/donor molar ratio** | ***E*, %** | ***E*_UiO-67-Ru(bpy)₃-_*_pse_*_,_ %** |
| --- | --- | --- | --- |
| 1 | 0.74 | 14 ± 1 | 9 ± 3 |
| 1*^a^* | – | 5 ± 1 | – |
| 2 | 0.89 | 15 ± 2 | 12 ± 2 |
| 2*^a^* | – | 3 ± 1 | – |
| 2*^b^* | 0.89 | 15 ± 3 | 12± 3 |
| 3 | 1.0 | 15 ± 3 | 12 ± 3 |
| 3*^a^* | – | 3 ± 1 | – |
| 4 | 1.5 | 15 ± 2 | 11 ± 3 |
| 4*^a^* | – | 4 ± 2 | – |

*^a^*the control experiment for the system in the absence of the acceptor

*^b^*photoluminescence quenching of PVK by UiO-67-Ru(bpy)_3_-*pse* after its use in the phenylboronic

acid photooxidation reaction

**
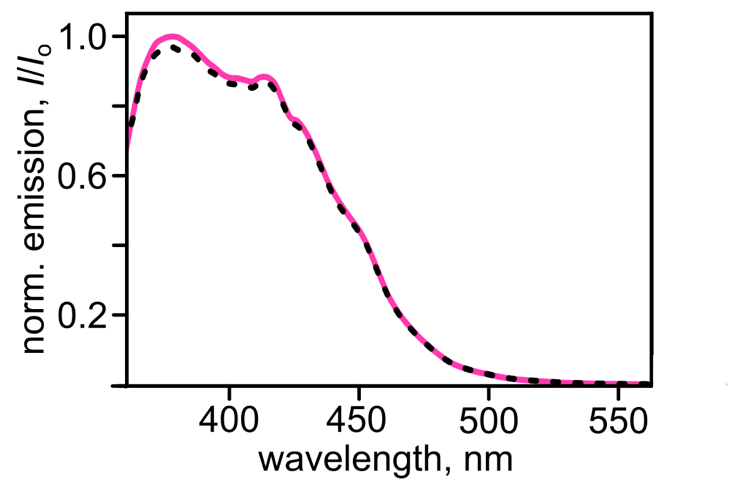
**

**Figure S22.** Emission spectra of PVK (solid pink line, *λ*_ex_ = 345 nm; 5.61 µM in DMF) and PVK in a UiO-67 suspension (*λ*_ex_ = 345 nm; dashed black line) with an acceptor-to-donor molar ratio of 0.89.


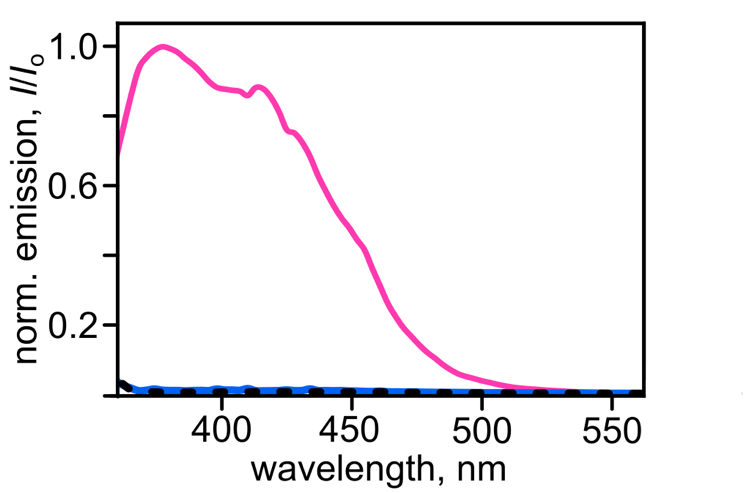


**Figure S23.** Emission spectra of PVK (solid pink line; *λ*_ex_ = 345 nm; 5.61 µM in DMF), UiO-67-Ru(bpy)_3_-*pse*, and UiO-67 (*λ*_ex_ = 345 nm, solid blue and dashed black lines).

**Table S7.** Summary of photoluminescence quenching of PVK in the presence of UiO-67-Ru(bpy)_3_-*dn* as a function of acceptor-to-donor molar ratio. Data were acquired from photoluminescence spectra collected in DMF.

| **entry** | **acceptor/donor molar ratio** | ***E*, %** | ***E*_UiO-67-Ru(bpy)₃-_*_dn_*_,_ %** |
| --- | --- | --- | --- |
| 1 | 2.7 | 9 ± 0.2 | 5 ± 2 |
| 1*^a^* | – | 4 ± 3 | – |
| 2 | 3.6 | 8± 2 | 5 ± 3 |
| 2*^a^* | – | 3 ± 1 | – |
| 2*^b^* | – | 6 ± 0.1 | 3± 1 |
| 3 | 4.1 | 8± 3 | 5 ± 3 |
| 3*^a^* | – | 3 ± 1 | – |
| 4 | 6.0 | 7 ± 2 | 5 ± 3 |
| 4*^a^* | – | 2 ± 0.4 | – |

*^a^*the control experiment for the system in the absence of the acceptor

*^b^*photoluminescence quenching of PVK by UiO-67-Ru(bpy)_3_-*dn* after its use in the phenylboronic

acid photooxidation reaction

**
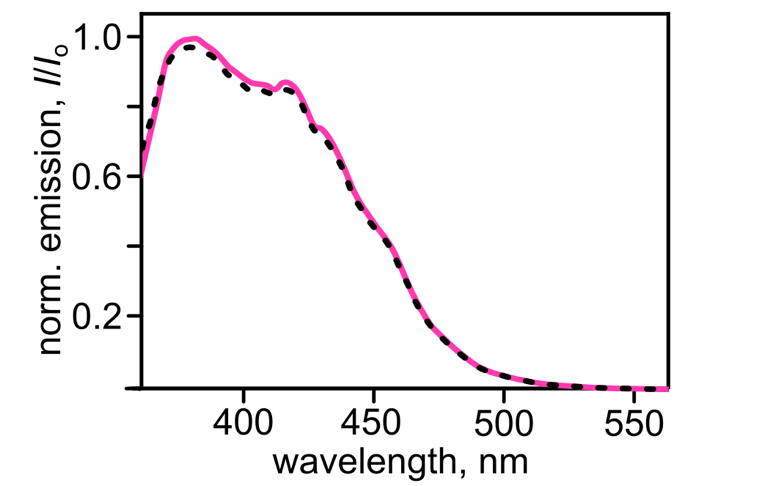
**

**Figure S24.** Emission spectra of PVK (solid pink line, *λ*_ex_ = 345 nm; 4.23 µM in DMF) and PVK in a UiO-67 suspension (*λ*_ex_ = 345 nm; dashed black line) with an acceptor-to-donor molar ratio of 3.6.

**
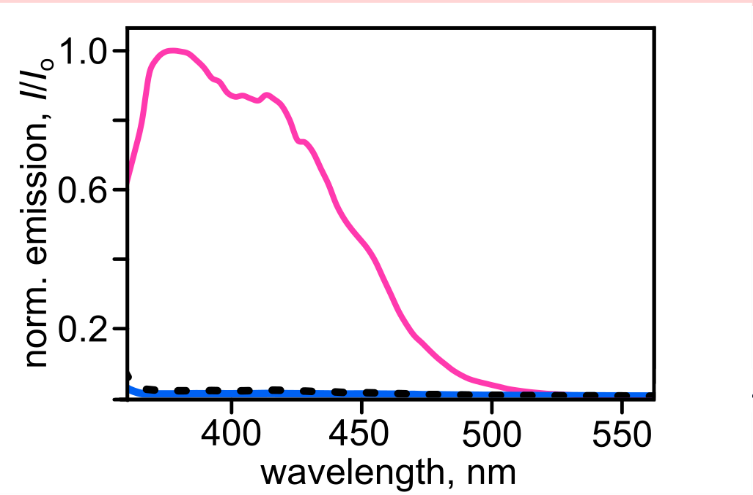
**

**Figure S25.** Emission spectra of PVK (solid pink line; *λ*_ex_ = 345 nm; 4.23 µM in DMF), UiO-67-Ru(bpy)_3_-*dn*, and UiO-67 (*λ*_ex_ = 345 nm, solid blue and dashed black lines).

**Table S8.** Summary of results for FRET measurements.

| **entry** | **system** | **acceptor/donor molar ratio*^a^*** | ***C*_catalyst_, wt %** | ***Q*_d_, %** | ***E*, %** |
| --- | --- | --- | --- | --- | --- |
| 1 | RuPNP@UiO-66 | 0.15 | 0.005 | 9.5 ± 1.7 | 36 ± 1 |
| 2 | HG2@UiO-66 | 0.19 | 0.059 | 11.9 ± 0.7 | 23 ± 4 |
| 3 | HG2@UiO-67 | 0.25 | 0.101 | 12.1 ± 1.9 | 14 ± 3 |
| 3*^b^* | HG2@UiO-67 | 0.25 | 0.084 | 12.1 ± 1.9 | 13 ± 3 |
| 4 | HG2@SBA-15 | 0.11 | 0.155 | 24.0 ± 1.3 | 13 ± 1 |
| 4*^c^* | HG2@SBA-15 | 0.11 | 0.104 | 24.0 ± 1.3 | 13 ± 2 |
| 5 | UiO-67-Ru(bpy)_3_-*pse* | 0.89 | 0.161 | 12.3 ± 0.2 | 12 ± 2 |
| 5*^d^* | UiO-67-Ru(bpy)_3_-*pse* | 0.89 | 0.168 | 12.3 ± 0.2 | 12 ± 3 |
| 6 | UiO-67-Ru(bpy)_3_-*dn* | 3.6 | 0.388 | 12.2 ± 0.2 | 5 ± 3 |
| 6*^d^* | UiO-67-Ru(bpy)_3_-*dn* | 3.6 | 0.268 | 12.2 ± 0.2 | 3 ± 1 |

*^a^*PVK (donor) moles were calculated using Mn = 23,000

*^b^*photoluminescence quenching of PVK by HG2@UiO-67 after its use in the RCM reaction *^c^*photoluminescence quenching of PVK by HG2@SBA-15 after its use in the ROMP reaction

*^d^*photoluminescence quenching of PVK by UiO-67-Ru(bpy)_3_-pse/*dn* after its use in the phenylboronic acid photooxidation reaction


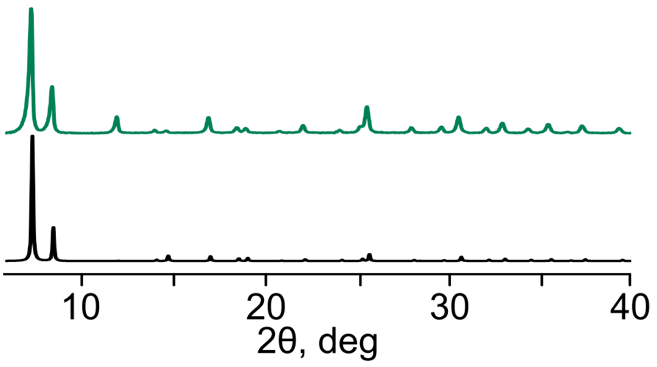


**Figure S26.** PXRD patterns of simulated UiO-66 (black),^[12]^ as well as RuPNP@UiO-66 (green).


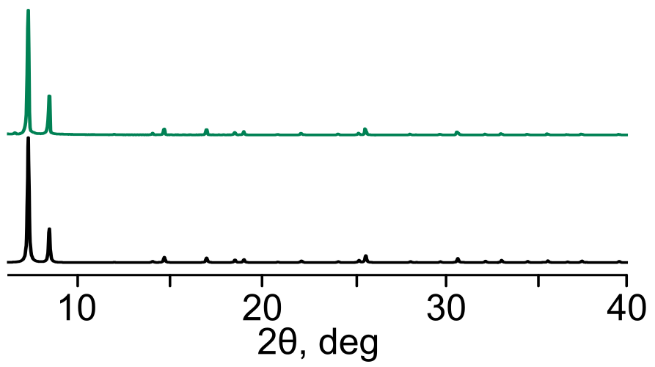


**Figure S27.** PXRD patterns of simulated UiO-66 (black),^[12]^ as well as HG2@UiO-66 (green).


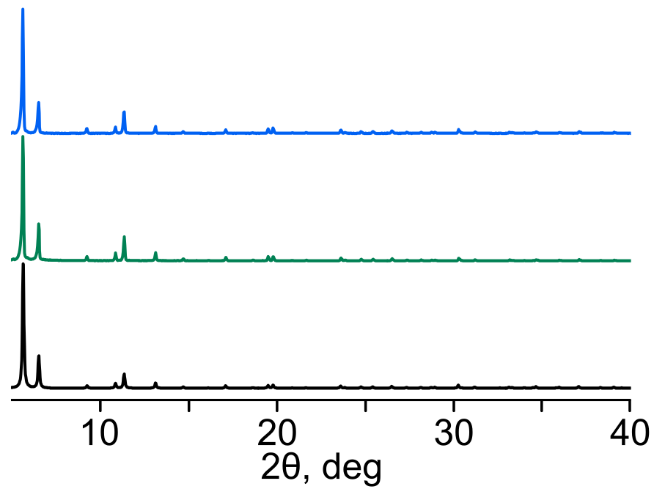


**Figure S28.** PXRD patterns of simulated UiO-67 (black),^[13]^ as well as HG2@UiO-67 before (green) and after (blue) its use in the RCM catalytic conditions.


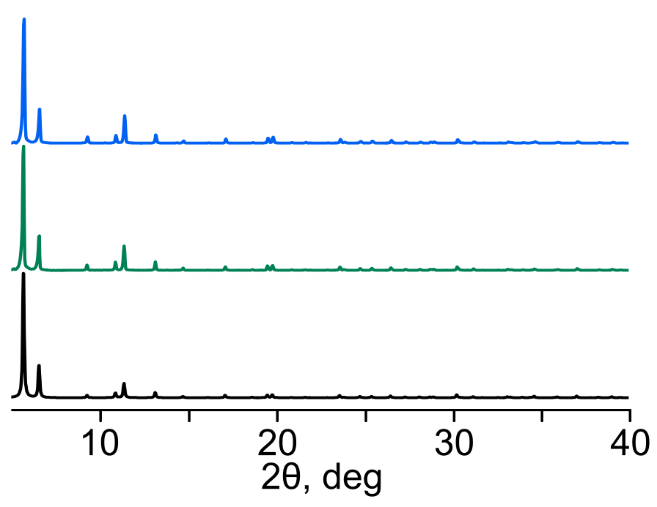


**Figure S29.** PXRD patterns of simulated UiO-67 (black),^[13]^ as well as UiO-67-Ru(bpy)_3_-*pse* before (green) and after (blue) its use in the photooxidation of phenylboronic acid.


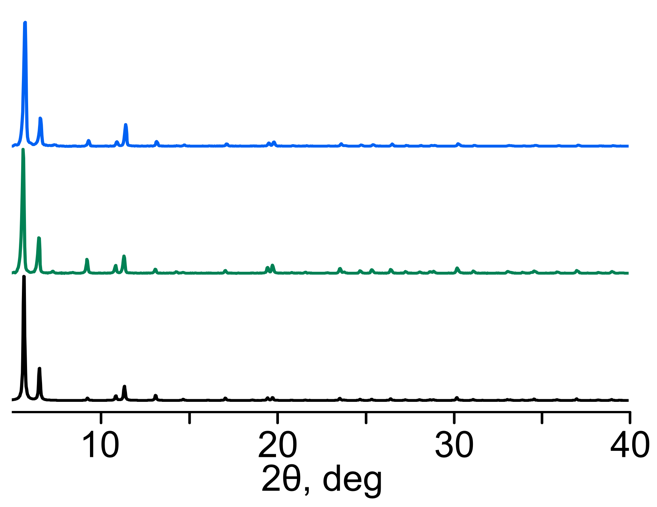


**Figure S30.** PXRD patterns of simulated UiO-67 (black),^[13]^ as well as UiO-67-Ru(bpy)_3_-*dn* before (green) and after (blue) its use in the photooxidation of phenylboronic acid.

**
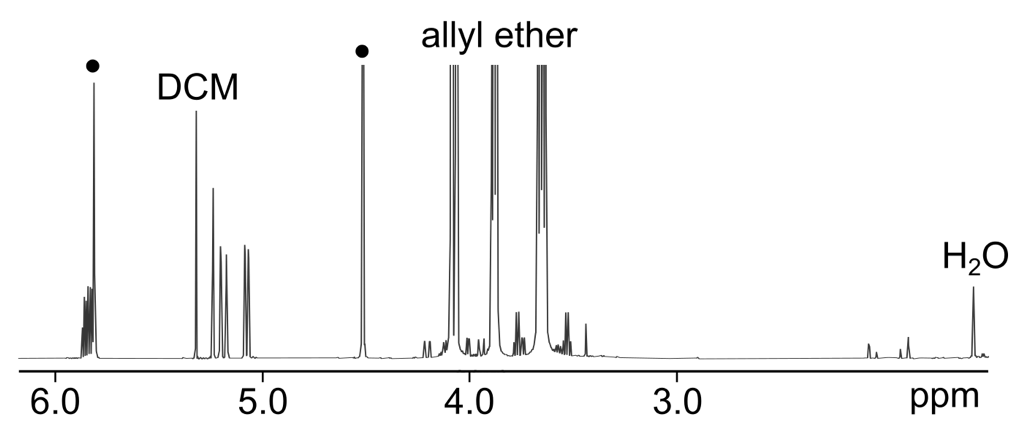
**

**Figure S31.** ^1^H NMR spectrum of crude product of the ring-closing metathesis using HG2@UiO-67 (Scheme 2). The resonances corresponding to the product, 2,5-dihydrofuran (●), are labeled.

**
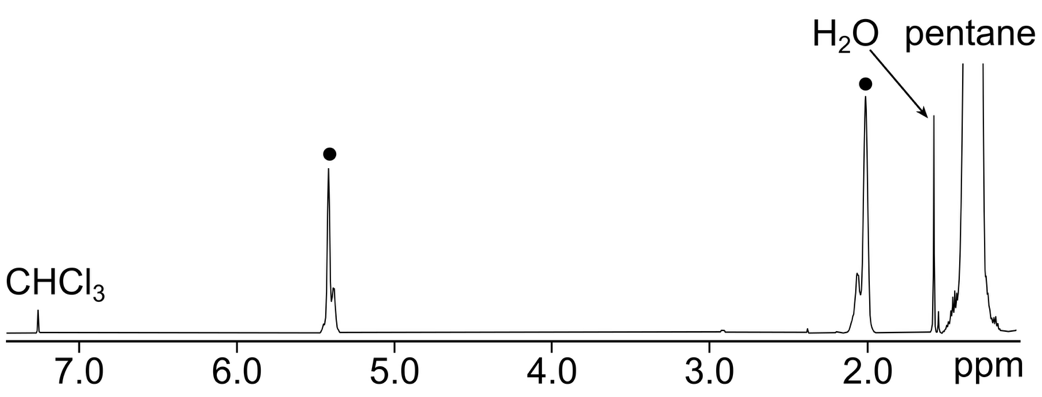
**

**Figure S32.** ^1^H NMR spectrum of crude product of the ring-opening metathesis polymerization using HG2@SBA-15 (Scheme 2). The resonances corresponding to the product, poly(cyclooctene) (●), are labeled.


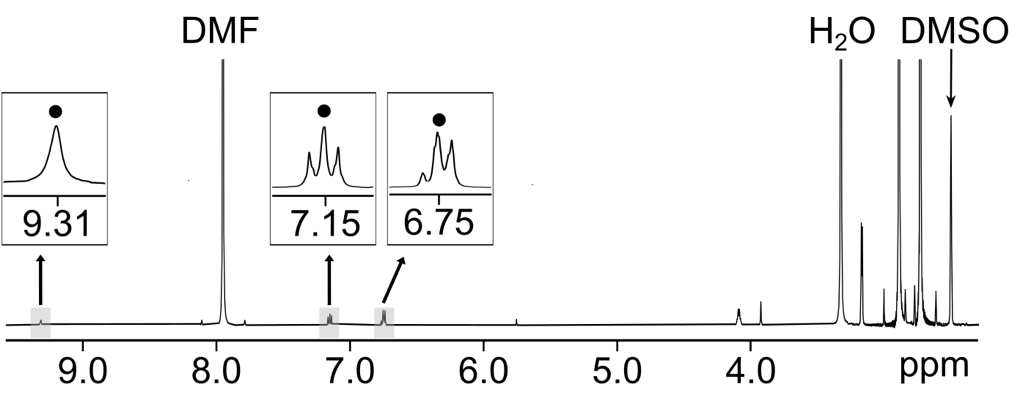


**Figure S33.** ^1^H NMR spectrum of crude product of the photooxidation of phenylboronic acid using UiO-67-Ru(bpy)_3_-*pse* (Scheme 2). The resonances corresponding to the product, phenol (●), are labeled.


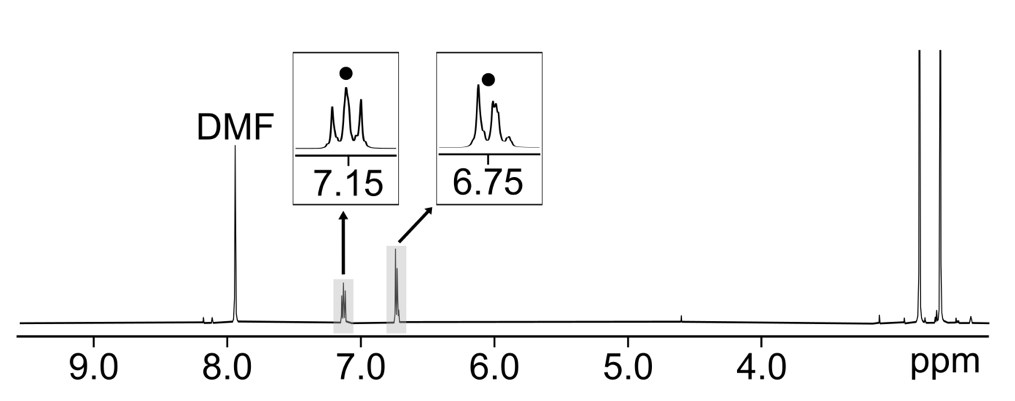


**Figure S34.** ^1^H NMR spectrum of crude product of the photooxidation of phenylboronic acid using UiO-67-Ru(bpy)_3_-*dn* (Scheme 2). The resonances corresponding to the product, phenol (●), are labeled.


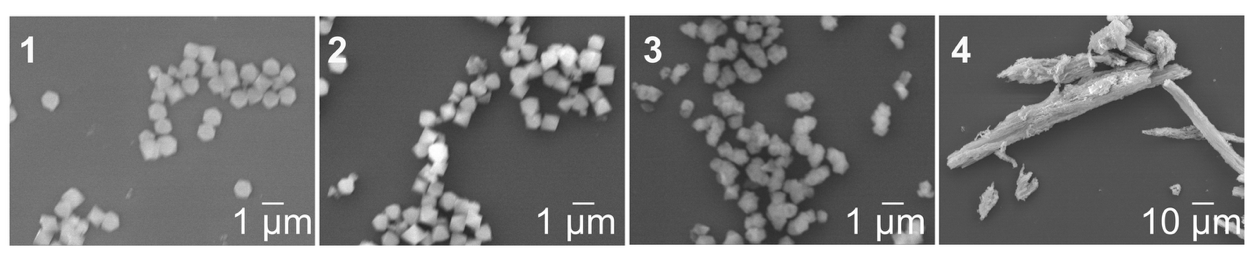


**Figure S35.** Scanning electron micrographs of catalyst@MOF/SBA-15 samples: (1) RuPNP@UiO-66, (2) HG2@UiO-66, (3) HG2@UiO-67, and (4) HG2@SBA-15.


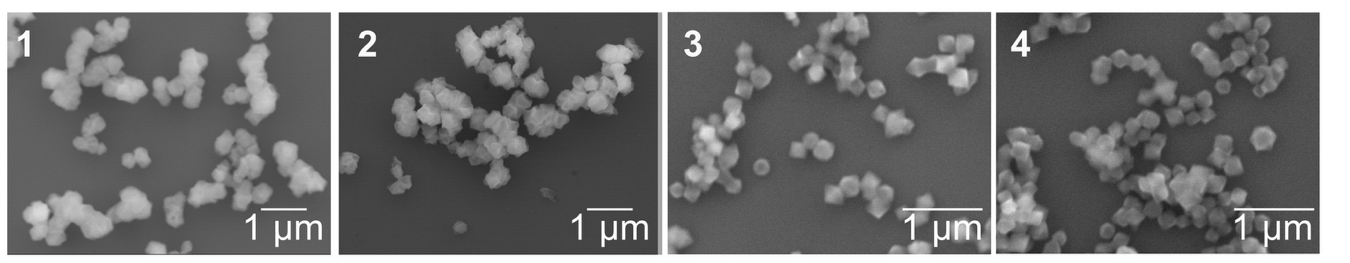


**Figure S36.** Scanning electron micrographs: (1) as-synthesized UiO-67-Ru(bpy)_3_-*pse*, (2) UiO-67-Ru(bpy)_3_-*pse* after its use in the photooxidation of phenylboronic acid, (3) as-synthesized UiO-67-Ru(bpy)_3_-*dn*, and (4) UiO-67-Ru(bpy)_3_-*dn* after its use in the photooxidation of phenylboronic acid.


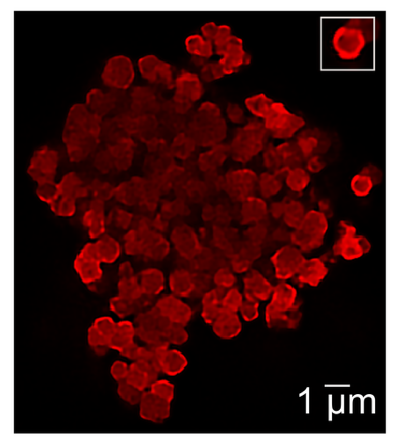


**Figure S37.** Confocal microscopy image of UiO-67-Ru(bpy)_3_-*pse* crystals showing fluorescence intensity across a single cross section taken along the *Z*-axis. The inset shows an image of a crystal selected from the cluster, demonstrating higher emission intensity along the periphery of the crystal (*λ*_ex_ = 460 nm).

# References

[1] B. J. Burger, J. E. Bercaw, “Chapter 4-Vacuum Line Techniques for Handling Air-Sensitive Organometallic Compounds.” in Experimental Organometallic Chemistry*, ACS Symposium Series,* **1987**, *357*, 79–115.

[2] B. Gnanaprakasam, J. Zhang, D. Milstein, *Angew. Chem. Int. Ed.* **2010**, *49*, 1468–1471.

[3] Z. Li, T. M. Rayder, L. Luo, J. A. Byers, C.-K. Tsung, *J. Am. Chem. Soc.* **2018**, *140*, 8082–8085.

[4] C. Wang, Z. Xie, K. E. deKrafft, W. Lin, *J. Am. Chem. Soc.* **2011**, *133*, 13445–13454.

[5] G. C. Shearer, S. Chavan, J. Ethiraj, J. G. Vitillo, S. Svelle, U. Olsbye, C. Lamberti, S. Bordiga, K. P. Lillerud, *Chem. Mater.* **2014**, *26*, 4068–4071.

[6] Y. Zhao, Q. Zhang, Y. Li, R. Zhang, G. Lu, *ACS Appl. Mater. Interfaces* **2017**, *9*, 15079–15085.

[7] Z. Zhou, Y. Wang, W.-S. Lo, G. J. Giardino, K. Lalit, M. Goldstein, W. Wang, C. Fields, A. Barney, C.-K. Tsung, U. Mohanty, W. Huang, J. Niu, *Nat*. *Commun*. **2025**,*16*, 8738.

[8] M. Bru, R. Dehn, J. H. Teles, S. Deuerlein, M. Danz, I. B. Müller, M. Limbach, *Chem. Eur. J.* **2013**, *19*, 11661–11671.

[9] X. Yu, S. M. Cohen, *Chem. Commun.* **2015**, *51*, 9880–9883.

[10] H. Balcar, T. Shinde, N. Žilková, Z. Bastl, *Beilstein J. Org. Chem.* **2011**, *7*, 22–28.

[11] J. R. Lakowicz, *Principles of fluorescence spectroscopy*, 3rd ed., Springer, New York, **2006**.

[12] L. Valenzano, B. Civalleri, S. Chavan, S. Bordiga, M. H. Nilsen, S. Jakobsen, K. P. Lillerud, C. Lamberti, *Chem. Mater.* **2011**, *23*, 1700–1718.

[13] N. Ko, J. Hong, S. Sung, K. E. Cordova, H. J. Park, J. K. Yang, J. Kim, *Dalton Trans.* **2015**, *44*, 2047–2051.

# Author contributions.

B. K. P. Maldeni Kankanamalage: writing of original draft (lead), writing - reviewing and editing (supporting), investigation (lead), methodology (lead), formal analysis (lead), and visualization (lead)

W. J. Thompson: writing - reviewing and editing (supporting), investigation (supporting), and formal analysis (supporting)

D. N. Smith: writing - reviewing and editing (supporting), investigation (supporting), visualization (supporting)

G. C. Thaggard: writing of original draft (supporting), writing - reviewing and editing (supporting), visualization (supporting)

N. Wijerathne: investigation (supporting), resources (supporting)

I. Incognito: investigation (supporting)

J. A. Byers: conceptualization (lead), funding acquisition (lead), project administration (lead), supervision (lead)

J. Niu: conceptualization (lead), funding acquisition (lead), project administration (lead), supervision (lead), resources (lead), writing - reviewing and editing (supporting)

N. B. Shustova: conceptualization (lead), funding acquisition (lead), project administration (lead), supervision (lead), resources (lead), formal analysis (lead), methodology (lead), writing of original draft (lead), writing - reviewing and editing (lead)
